# Isolation, Structure Determination, and Synthesis of Cyclic Tetraglutamic Acids from Box Jellyfish Species *Alatina alata* and *Chironex yamaguchii*

**DOI:** 10.3390/molecules25040883

**Published:** 2020-02-17

**Authors:** Justin Reinicke, Ryuju Kitatani, Shadi Sedghi Masoud, Kelly Kawabata Galbraith, Wesley Yoshida, Ayako Igarashi, Kazuo Nagasawa, Gideon Berger, Angel Yanagihara, Hiroshi Nagai, F. David Horgen

**Affiliations:** 1 Department of Natural Sciences, Hawaii Pacific University, Kaneohe, HI 96744, USA; reinicke@hawaii.edu (J.R.); kgalbraith2468@gmail.com (K.K.G.); 2 Daniel K. Inouye College of Pharmacy, University of Hawaii at Hilo, Hilo, HI 96720, USA; 3 Department of Marine Sciences, Tokyo University of Marine Science and Technology, Tokyo 108-8477, Japan; ryujukitatani@gmail.com (R.K.); igarashi.b.ayako.t@gmail.com (A.I.); 4 Department of Biotechnology and Life Science, Tokyo University of Agriculture and Technology, Tokyo 184-8588, Japan; shadi@m2.tuat.ac.jp (S.S.M.); knaga@cc.tuat.ac.jp (K.N.); 5 German Center for Neurodegenerative Diseases (DZNE), Sigmund-Freud-Str. 27, 53127 Bonn, Germany; 6 Department of Chemistry, University of Hawaii at Manoa, Honolulu, HI 98622, USA; wyoshida@hawaii.edu; 7 Békésy Laboratory of Neurobiology, Pacific Biosciences Research Center, School of Ocean and Earth Science and Technology, and Department of Tropical Medicine, John A. Burns School of Medicine, University of Hawaii at Manoa, Honolulu, HI 96822, USA

**Keywords:** cubozoa, cnidarian, cnidarin, cyclicpeptide, polyglutamic acid, venom

## Abstract

Cubozoan nematocyst venoms contain known cytolytic and hemolytic proteins, but small molecule components have not been previously reported from cubozoan venom. We screened nematocyst extracts of *Alatina alata* and *Chironex yamaguchii* by LC-MS for the presence of small molecule metabolites. Three isomeric compounds, cnidarins 4A (**1**), 4B (**2**), and 4C (**3**), were isolated from venom extracts and characterized by NMR and MS, which revealed their planar structure as cyclic γ-linked tetraglutamic acids. The full configurational assignments were established by syntheses of all six possible stereoisomers, comparison of spectral data and optical rotations, and stereochemical analysis of derivatized degradation products. Compounds **1**–**3** were subsequently detected by LC-MS in tissues of eight other cnidarian species. The most abundant of these compounds, cnidarin 4A (**1**), showed no mammalian cell toxicity or hemolytic activity, which may suggest a role for these cyclic tetraglutamates in nematocyst discharge.

## 1. Introduction

### Background

Jellyfish, sea anemones, hydrozoans, and corals comprise the phylum Cnidaria. The phylum is defined by the presence of stinging cells, cnidocytes, which contain a singular large subcellular collagen-walled organelle, the cnidae, capable of rapid discharge of a hollow eversible tubule. Penetrant cnidae are called nematocysts, which store venom that is injected into prey upon discharge through the tubule. Both physical and chemical stimuli trigger nematocyst discharge.

Cnidarians in the class Cubozoa, or box jellyfish, include about 50 described species [1] that can cause harm and even death to humans upon envenomation. For example, *Chironex fleckeri* has caused more than 67 cardiac/respiratory related human fatalities [2,3,4,5]. In general, envenomation symptoms from cubozoans range from immediate localized pain and swelling, to deferred systemic responses, such as Irukandji syndrome, which is characterized by delayed symptoms including nausea, vomiting, headaches, muscle pain, pulmonary edema, hypertension and cardiac failure [6,7,8,9]. *Chironex yamaguchii*, known as the Habu-kurage in Japan, is closely related to *Chironex fleckeri* [10] and also has confirmed human fatalities associated with its sting. However, the majority of *C. yamaguchii* stings are not fatal [11]. The Hawaiian Box Jellyfish, *Alatina alata* [1,12] causes stings that are typically characterized by localized pain and welts [9,13]. However, there have also been cases of systemic Irukandji sequelae associated with *Alatina alata* stings. Yoshimoto and Yanagihara retrospectively identified symptoms consistent with systemic Irukandji symptoms in 5% of patients [14]. The mechanisms of venom induced Irukandji systemic symptoms are not clearly understood but have been found to involve induction of “cytokine storm” and “catecholamine surge” events comparable to sepsis [7,8,9].

The major reported toxins isolated from cubozoan venom are proteins that demonstrate hemolytic and cytolytic activity [15,16,17,18,19], and some are allergenic [11]. Recent proteomic approaches have revealed a more complex composition of venom protein families [20,21,22]. For species lethal to humans, cardiovascular collapse appears to be the common biological outcome, but the mechanisms of toxicity may be complex and vary among cubozoa species [3,19,23,24,25,26,27,28]. Controversy exists as to whether cytolytic proteins, porins, may be solely responsible for cardiovascular collapse and systemic effects observed in humans [3,19,23,24,25,26,27,29]. Further complicating analysis of the published literature is the vast differences in reported specific activity of “venom” and the methodologies by which “venom” is prepared.

Some groups of cnidarians, especially members of Anthozoa (corals and anemones), are rich sources of bioactive secondary metabolites that putatively serve in defense and communication, and have potential societal benefits such as potential as therapeutic drugs [30]. However, few small molecules have been reported from scyphozoans (true jellyfish), and none from cubozoans [31]. At the same time, there is evidence for small molecules in some cnidarian venoms. Lindquist reported tridentatols, alkylthio tyramine derivatives, from the hydroid *Tridentata marginata* [32]. This suggests the potential of small molecules in other cnidarian venom, including box jellyfish venom.

**Figure 1:**
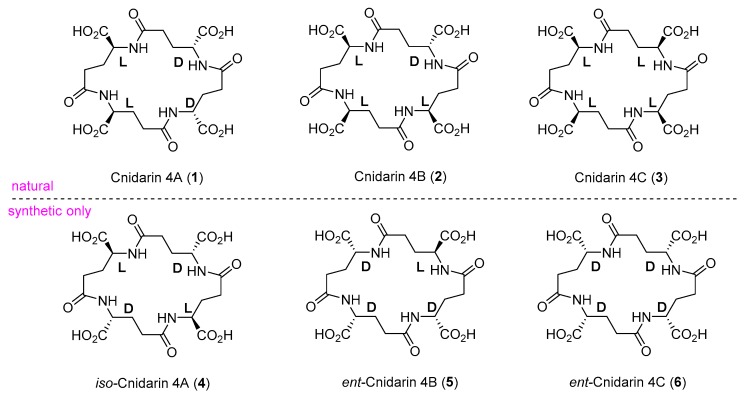
Structure of naturally occurring cyclic tetra-γ-glutamic acids **1**–**3** and synthetic analogues **4**–**6**.

In this study, Hawaiian box jellyfish, *Alatina alata*, and Japanese box jellyfish, *Chironex yamaguchi* (Habu-kurage), were selected as model species to expand the known chemical space of venom constituents by screening for small molecules. Venom extracts from both *A. alata* and *C. yamaguchi* yielded cnidarin 4A (**1**), 2.1,5.4-anhydro(γ-d-glutamyl-γ-d-glutamyl-γ-l-glutamyl- l-glutamic acid), and stereoisomers cnidarins 4B (**2**) and 4C (**3**), (dlll and llll analogs, respectively) were isolated from *C. yamaguchii* (Figure 1). The structures of **1**–**3** were determined by spectroscopic analyses, chemical synthesis, and analysis of chemical degradation products. Cnidarins 4A (**1**) and 4B (**2**) are novel natural products, while cnidarin 4C (**3**) has been synthesized previously but is reported here for the first time from nature. To our knowledge, these are the first small molecules isolated and fully characterized from Cubozoa.

## 2. Results

### 2.1. Alatina alata Venom

*Alatina alata* specimens were collected on Waikiki Beach (Oahu, Hawaii). After gentle removal of nematocysts from excised tentacles, nematocysts were disrupted and venom was collected from supernatant after centrifugation (details provided in Section 4.2.1 and Section 4.3.1). LC-MS screening of the venom extracts (Figure S1, R_t_ 18.6 min) consistently showed a constituent demonstrating a protonated molecular ion at *m*/*z* 517.1765, which supported a molecular formula of C_20_H_28_N_4_O_12_ (C_20_H_29_N_4_O_12_^+^ requires *m*/*z* 517.1783, ∆ −1.8 mmu) and indicating 9 degrees of unsaturation. The peak was collected by analytical LC guided by MS, and the ^1^H-NMR of the resulting enriched sample of cnidarin 4A (**1**) showed signals consistent with the presence of glutamic acid residues, while the molecular formula matched a tetraglutamate with one additional degree of unsaturation. A detailed NMR study (HSQC, HMBC, NOESY, and 1-D TOCSY) confirmed the presence of glutamic acid residues, while NOESY correlations between NH and γ-protons (and lack of NH to α-protons) was evidence of all γ-linkages. The data suggested cnidarin 4A (**1**) was a cyclic γ-linked tetraglutamate. 

Following further purification of cnidarin 4A (**1**) (Figure S2, R_t_ 9.4 min), the configuration of the glutamic acid residues in **1** was determined by the advanced Marfey’s analysis following acid hydrolysis. The resulting derivatized glutamate residues were quantitated by both UV and MS/MS, and showing that cnidarin 4A (**1**) contains a 1:1 ratio of d-glutamic acid:l-glutamic acid (see Table S1 and Figures S4 and S5). 

**Scheme 1:**
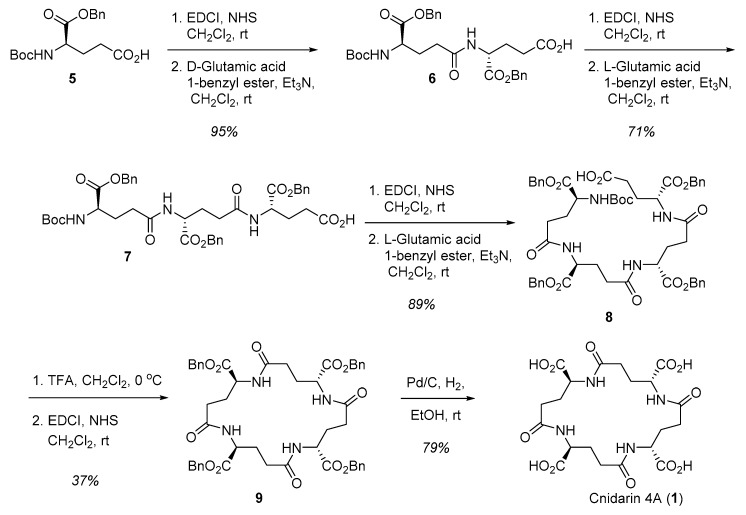
Preparation synthetic cnidarin 4A (**1**).

Taken together, the data suggested that the isolated compound was a cyclic tetrapeptide of glutamic acids with a 1:1 ratio of d/l and all γ-linked. Only two possible cyclic tetra-γ-glutamates meet this criteria, having a configuration sequence of either ddll or dldl. In order to determine the structure and obtain additional amounts of the scarce natural product for biological testing, both compounds, 2.1,5.4-anhydro(γ-d-glutamyl-γ-d-glutamyl-γ-l-glutamyl-l-glutamic acid) (cnidarin 4A, **1**) and 2.1,5.4-anhydro(γ-d-glutamyl-γ-l-glutamyl-γ-d-glutamyl-l-glutamic acid) (*iso*-cnidarin 4A, **4**), were synthesized. The syntheses were performed similar to methods described by Munekata and coworkers [33] and Hollosi and Kajtar [34] who coincidently synthesized the cyclic tetrapeptide with γ-linkage containing all l-glutamic acid residues, designated below as cnidarin 4C (**3**). For the ddll-cyclotetra-γ-glutamic acid, cnidarin 4A (**1**), Boc-d-glutamic acid 1-benzyl ester (**5**) was converted to the *N*-hydroxysuccinimide (NHS) ester and then coupled with d-glutamic acid 1-benzyl ester in dichloromethane with triethylamine, to provide protected dipeptide **6** (Scheme 1). 

This process of NHS acyl activation, followed by coupling, was repeated two more times, using the protected l-glutamate, with comparable yields, to give the linear tetrapeptide 8. Trifluoroacetic acid (TFA) was used to remove the *tert*-butoxycarbonyl (Boc) protecting group, providing the free ammonium trifluoroacetate, primed for cyclization. Macrocyclization was achieved using a carbodiimide to yield 9 in adequate yield. Benzyl deprotection of the alpha carboxylic acids was accomplished using standard catalytic hydrogenation conditions to provide crude cnidarin 4A (**1**). The crude product was purified by reverse phase HPLC to provide 2.1,5.4-anhydro (γ-d-glutamyl-γ-d-glutamyl-γ-l-glutamyl-l-glutamic acid), cnidarin 4A (**1**), in nine steps (18.5% overall yield). The synthesis of the dldl-cyclotetra-γ-glutamate isomer, *iso*-cnidarin 4A (**4**), was performed via the same approach (see Supplemental Information).

**Figure 2:**
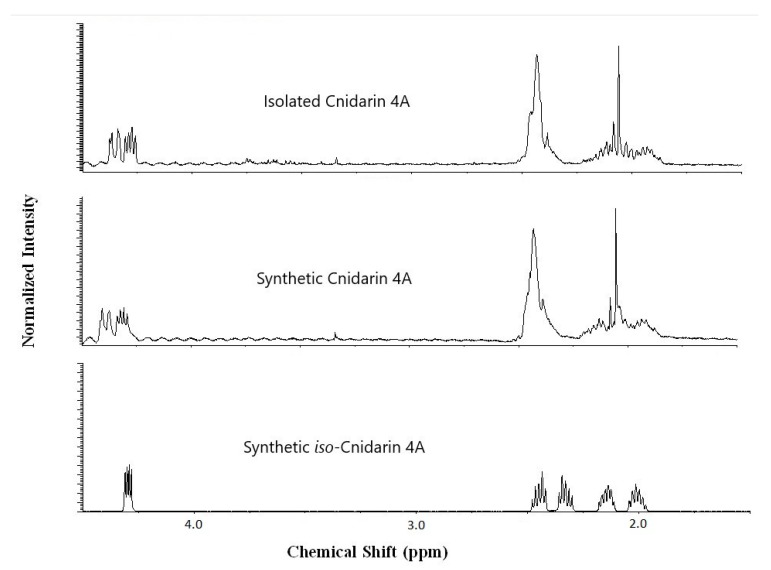
^1^H-NMR of cnidarin 4A (**1**) vs. synthetic samples of cnidarin 4A (**1**) and *iso*-cnidarin 4A (**4**) (300 MHz; **1** in D_2_O/100 mM deuterated formic acid and **4** in D_2_O).

^1^H-NMR of the venom isolate cnidarin 4A (**1**) showed evidence of 2 sets of glutamate signals that matched that of synthetic product 2.1,5.4-anhydro(γ-d-glutamyl-γ-d-glutamyl-γ-l-glutamyl-l-glutamic acid) (cnidarin 4A, **1**), but differed significantly from *iso*-cnidarin 4A (**4**) (Figure 2). To confirm, the tetramethyl esters of isolated **1** and synthesized **1** were prepared by Fischer esterification in methanol, catalyzed by Amberlyst-15. The ^13^C-NMR spectra of both resulting tetramethyl esters showed the expected 12 carbons signals with nearly identical chemical shifts (Table 1). Thus, **1** was unambiguously identified as the all γ-linked cyclic tetraglutamate with a ddll configuration. 

### 2.2. Chironex Yamaguchii Venom

**Figure 3:**
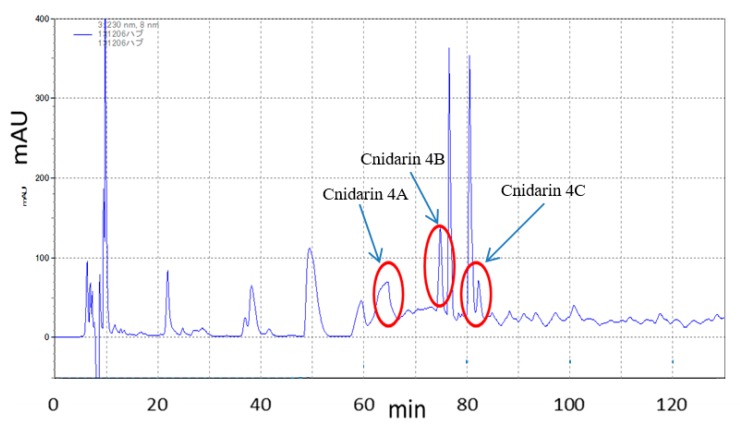
Reversed phased HPLC chromatogram of *Chironex yamaguchii* nematocyst extracts using Develosil C30 column. Cnidarin 4A (**1**), 4B (**2**), and 4C (**3**) peaks are indicated. Detection is by UV.

The venom of *Chironex yamaguchii* was similarly screened for secondary metabolites. Cnidarin 4A (**1**) and two additional isomers were isolated from nematocyst extracts. The peptides were isolated guided by LC-MS monitoring [M + H]^+^ *m*/*z* 517 (or [M − H]^−^ *m*/*z* 515), as cnidarin 4A (**1**), cnidarin 4B (**2**), and cnidarin 4C (**3**), in order of HPLC elution (Figure 3). As in *Alatina alata*, cnidarin 4A (**1**) was the major cyclic tetraglutamate. Cnidarins 4B (**2**) and 4C (**3**) also have a molecular formula of C_20_H_28_N_4_O_12_ (cnidarin 4B (**2**): [M + H]^+^ at *m*/*z* 517.1780, ∆ −0.3 mmu; cnidarin 4C (**3**): [M + H]^+^ at *m*/*z* 517.1775 ∆ −0.8 mmu).

The ^1^H-NMR spectra of cnidarins 4A (**1**), 4B (**2**), and 4C (**3**) showed close similarity. The complete structure elucidation of cnidarin 4B (**2**) and 4C (**3**) was accomplished by the comparison with all six of the possible stereoisomers (**1**–**6**) that were synthesized in this study.

**Scheme 2:**
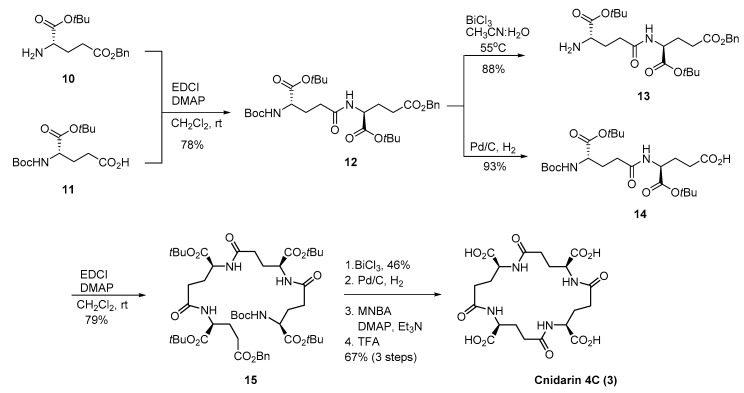
Preparation cnidarin 4C (**3**).

The high degree of symmetry in the llll-cyclotetra-γ-glutamic acid (**3**) lent itself to a second synthetic strategy (Scheme 2). l-Glutamic acid 5-benzyl ester **10** was reacted with Boc-l-glutamic acid (**11**) to give protected ll-glutamylglutamic acid (**12**). The ll-glutamylglutamic acid derivatives **13** and **14** were synthesized from **12** by deprotection of the benzyl group and selective deprotection of the Boc, respectively. Compound **13** was reacted with **14** to give the protected linear llll-tetra-γ-glutamic acid **15**. Deprotection of Boc and the benzyl group was followed by macrocyclization using MNBA/DMAP. Finally, TFA was used to remove the *tert*-butyl protecting group, providing cnidarin 4C (**3**) in nine steps (18.5% overall yield/LLS). The dddd-configured enantiomer, *ent*-cnidarin 4C (**6**), was similarly prepared.

The ^1^H and ^13^C-NMR spectra of authentic cnidarian 4C (**3**) were identical with those of the synthesized llll- and dddd-configured cyclotetra-γ-glutamic acids (**3** and **6**, respectively). The optical rotation of these compounds was small and difficult to compare with the natural product given its scarcity. Therefore, the Marfey analysis was used to determine the configuration of glutamic acid residues in cnidarin 4C (**3**). Following hydrolysis of the natural product, only l-glutamic acid was detected indicating that cnidarin 4C (**3**) is the llll-configured cyclic tetra-γ-glutamic acid.

In order to elucidate the absolute structure of cnidarin 4B (**2**), the synthesis of the remaining 2 stereoisomers, the lddd- and dlll-configured enantiomers (**2** and **5**, respectively), was performed using the same approach as for synthesis of the llll- and dddd-enantiomeric pair, **3** and **6** (see Supplemental Information Section S1). The ^1^H and ^13^C-NMR spectra of authentic cnidarin 4B (**2**) matched with those of the synthesized lddd- and dlll-configured cyclotetra-γ-glutamic acids. Both natural cnidarin 4B (**2**) and the synthesized dlll isomer showed dextrorotatory optical rotation [cnidarin 4B (**2**), ${\left[\alpha \right]}_{D}^{24}$ + 30° (c 0.009, CH_3_OH); synthesized dlll isomer, ${\left[\alpha \right]}_{D}^{24}$ + 7.5° (c 0.022, CH_3_OH)], while synthesized lddd-configured *ent*-cnidarin 4B (**5**) showed levorotatory optical rotation, ${\left[\alpha \right]}_{D}^{24}$ −17° (c 0.022, CH_3_OH). Consequently, the structure of cnidarin 4B (**2**) was assigned as the dlll-configured cyclotetra-γ-glutamic acid. 

The ^1^H and ^13^C-NMR assignments for compounds **1**–**3** are shown in Table 2, Table 3 and Table 4, respectively.

### 2.3. Tissue Distribution of Cnidarins

To determine whether cnidarin 4A (**1**) and related isomers, occurred in the nematocyst only or also in adjacent tissues, *Alatina alata* tentacle extract, free of nematocysts, was analyzed by LC-MS. Neither cnidarin 4A (**1**) nor its isomers were detected by LC-MS (Figure S7), indicating that the cnidarin 4′s only occur in the nematocysts. LC-MS quantification of cnidarin 4A (**1**) in *Alatina alata* venom showed a concentration in extracted nematocyst venom of 5.7 μg/mL (±0.2, 4% CV).

### 2.4. Marine Animals Screened for Cnidarin 4A-C (***1**–**3***)

Seventeen marine animal species, including species from Cnidaria, Ctenophora, Annelida, Mollusca, Echinodermata, Arthropoda, and Chordata, were screened for cnidarin 4A–C (**1**–**3**) by LC-MS. Compounds **1**–**3** were detected in whole bodies or tentacles of all 9 cnidarians examined (including *C. yamaguchii*). However, none were detected in the 8 non-cnidarian species. These results suggest cnidarins 4A–C (**1**–**3**) are specific to cnidarians. 

### 2.5. Cytotoxicity and Hemolytic Activity

The cytotoxicity of cnidarin 4A (**1**) and *iso*-cnidarin 4A (**4**) was evaluated in HEK-293 (human embryonic kidney) cells. The compounds showed no significant toxicity at concentrations as high as 100 µM (Figure S9). This data suggests that cnidarin 4A (**1**) and *iso*-cnidarin 4A (**4**) are not directly toxic to human cells. The ability of cnidarin 4A (**1**) to lyse red blood cells was also measured at concentrations as high as 121 μM, but compound **1** had no effect on cell lysis. 

## 3. Discussion

In this study, we isolated and identified three cyclotetra-γ-glutamic acids, cnidarins 4A–C (**1**–**3**), from cubozoan nematocyst venom and determined that the compounds are distributed across numerous cnidarian taxa, while absent from animals in six other phyla. Structural analyses indicated that compounds **1**–**3** have all γ-linkage, and **1** and **2** contain D-glutamic acid residues. The full structural assignments were made by spectral, optical rotation, and degradation/configurational analyses in comparison with synthetic isomers **1**–**6**. Compounds **1**–**3** represent the first fully characterized small molecules reported from *A. alata* and *C. yamaguchii*. In fact, to our knowledge, **1**–**3** are the first characterized small molecules reported from the class Cubozoa. Interestingly, ctenophores were formerly grouped with cnidarians in the phylum Coelenterata, but increasing awareness of their differences resulted in their placement in a separate phylum. In this study, cnidarin 4A–C (**1**–**3**) were not detected in the Ctenophora *Bolinopsis mikado* but were detected in all cnidarian species tested, providing chemotaxonomic evidence that supports the re-classification of ctenophores separate from the cnidarians.

Although new in nature, the synthesis of compound **3** has been described previously [33,34] and was reported to be non-toxic in rats [33]. Cnidarins 4A–C (**1**–**3**) are closely related to poly-γ-glutamates (consisting of 10–50 glutamic acid residues) that have been reported as a major component of nematocysts in cnidarians [35,36] and are biosynthesized in nematocysts [37]. The resulting osmotic pressure of the polyanions contributes to the driving force behind the discharge of nematocysts [35,38,39]. Interestingly, poly-γ-glutamates are produced rarely by eukaryotes, and almost exclusively by Gram-positive bacteria (especially *Bacillus* species). Poly-γ-glutamates are non-toxic, non-immunogenic, and even edible, with numerous commercial applications including as food additives, drug carriers, cryoprotectants, and wastewater flocculants. 

This investigation did not elucidate a biological role for compounds **1**–**3**. Synthetic cyclotetraglutamic acids **1** and **4**, however, are not toxic to human cells in culture, and compound **1** showed no ability to cause hemolysis of red blood cells. The lack of cytotoxicity and hemolytic activity observed from compound **1** does not preclude a role in the bioactivity of the venom. However, it is quite possible that compounds **1**–**3** contribute to the nematocyst discharge and the lack of biological effects in our assays is consistent with such a role. Clearly, the wide distribution in Cnidaria suggest compounds **1**–**3** play some important role in nematocyst function or venom toxicity. Further studies will be required to determine the functional role of the cnidarin 4′s. 

## 4. Materials and Methods 

### 4.1. General Experimantal Procedures

LC-MS and MS were performed using either (a) an Agilent MSD-TOF time of flight (TOF) analyzer, (b) a Bruker micrOTOF QII mass spectrometer, or (c) a Thermo Finnigan LCQ Deca XP Max ion trap mass spectrometer with evaporative light scattering (ELSD, Sedex 75, Sedere, Alfortville, France) and photo diode array (PDA, Surveyor, Thermo Finnigan, San Jose, CA, USA) detectors, or (d) a JEOL JMS-T100LC spectrometer.

HPLC analyses and separations were performed using either a SHIMADZU HPLC system equipped with a SPD-M10A diode array detector or an Agilent 1100 binary preparative system equipped with a fraction collector (FC) and multi-wavelength UV detector. 

NMR experiments were performed on either (a) a Varian Unity INOVA 500 spectrometer using 3 mm tubes or a Protasis capillary probe, (b) a Varian Mercury Plus 300 MHz spectrometer using 3 and 5 mm tubes, (c) Bruker AVANCE III 800 MHz and 600 MHz spectrometers, or (d) JEOL AL300 and ECX 400 instruments. The spectra are referenced internally according to the residual solvent signals of CDCl_3_ (δ_H_ 7.26 ppm; δ_C_ 77.0 ppm), D_2_O (δ_H_ 4.79 ppm; 1,4-dioxane was added as internal standard for ^13^C-NMR, δ_C_ 67.2 ppm), CD_3_OD (δ_H_ 4.87 ppm), and d_6_-DMSO (methyl carbons δ_C_ 39.51 ppm). 

Optical rotations were measured on a JASCO P-2100 or P-2200 polarimeters. Infrared spectra were obtained with the use of NaCl and AgCl plates on a Perkin Elmer Spectrum RX I FT-IR. Flash chromatography was carried out on silica gel (Silicycle, SiliaFlash P60, 230−400 mesh, or Kanto silica gel 60 spherical, particle size 0.040–0.100 mm). Silica thin layer chromatography (TLC) plates (Sigma-Aldrich, St. Louis, MO, USA) were used to visualize reaction products, either under a UV lamp (254 nm) or with *para* -anisaldehyde stain. 

Starting materials H-Glu-OtBu·HCl and H-d-Glu-OtBu·HCl were obtained commercially (Watanabe Chem. Ind., Ltd., Hiroshima, Japan).

### 4.2. Species Sample Collection

#### 4.2.1. *Alatina alata*

Specimens were collected at Waikiki Beach (Oahu, HI, USA) 8–10 days after the full moon from 2007–2011. Organisms were collected immediately after beaching using metal tongs, and tentacles were immediately excised from the animals, transferred in to 1 M trisodium citrate solution (1 volume tentacle:4 volumes 1 M trisodium citrate) and transported to the lab. Specimens were identified by A.Y.

#### 4.2.2. *Chironex yamaguchii*

Specimens were collected at Irijima, Urasoe, Okinawa in August from 2006–2015. The samples were identified by Professor S. Kubota, Faculty of Science, Kyoto University. Tentacles were excised from the samples at the site immediately after collection, kept in the cold seawater and stored at −30 °C until treatment. The voucher specimens for identification were deposited at the Okinawa Prefectural Institute of Health and Environment.

### 4.3. Venom Extraction and Preparation

#### 4.3.1. *Alatina alata*

The buffered tentacles solution was gently rocked at 4 °C for 3–6 weeks to separate the nematocyst from tentacle tissue with a 90% nematocyst recovery. Sieved (0.5-mm mesh) nematocyst solutions were centrifuged at 400× *g* for 20 min. Undischarged nematocyst pellets were resuspended in chilled 1 M citrate at 1:20 (vol:vol) and washed twice at 250× *g* for 20 min, then gently diluted 1:0.5 (vol:vol) with ice-cold deionized water to a slurry and transferred to a pre-chilled French Press 20 K pressure cell (SLM-AMINCO Cat# FA078) and subjected to 12,000 psi for 10–15 min. The lysate (total venom) was expelled at 30 drops/min and recycled 2–4 times to achieve 90% nematocyst rupture, then centrifuged at 12,000× *g* at 4 °C for 5 min to remove structural capsule wall and tubule debris. The viscous upper layer, lysate (venom) was removed, leaving the solid debris pellet, aliquoted, snap frozen in N_2_(l) and stored at −80 °C. Protein concentrations were determined using a Bradford protein assay (Bio-Rad Protein Assay). Size-exclusion chromatography (SEC) was performed using a BioSilect 125-5 column (BioRad 125-0060 with BioRad 125-0072, Hercules, CA, USA) equilibrated with sodium phosphate buffer (50 mM Na_2_HPO_4_, 50 mM NaH_2_PO_4_, 150 mM NaCl, pH 6.8) at a rate of 0.5 mL/min using an AKTA Purifier high-pressure liquid chromatography (HPLC) system (GE Biosciences). Protein-free fractions (mw < 2000) of the extract were screened by LC-MS for small molecule components (Section 4.4.1).

Prior to analysis of tentacles, tentacles were examined microscopically and found to be devoid of nematocysts after incubation for weeks in 1 M citrate at 4 °C, allowing nematocyst sloughing. Tentacles were then freeze dried for solvent extraction and analysis by LC-MS to check for the presence of cnidarins 4A–C (**1**–**3**).

#### 4.3.2. *Chironex yamaguchii*

The tissue of the entire tentacles was used to isolate the nematocysts from *C. yamaguchii*. The tentacles used for experiments were excised from frozen tentacles. A string of tentacle was shaken vigorously for 5 min in 20 mL 1 M NaCl solution to isolate the nematocyst. NaCl solutions containing nematocyst (nematocyst suspension) were weakly centrifuged (100 rpm) and the isolated nematocysts were obtained as the pellet. The tentacles that were separated from nematocysts were used as the nematocyst free tentacles. The isolated nematocysts and nematocyst free tentacles were sonicated in distilled water. After centrifugation (12,000× *g*, 30 min), each supernatant was removed and analyzed by LC-MS.

### 4.4. Isolation of Compounds

#### 4.4.1. Isolation of Cnidarin 4A (**1**) from *Alatina alata*

Venom extract SEC fractions from *Alatina alata* were fractionated in multiple batches using HPLC (Waters Atlantis dC18, 10 × 250 mm, 10 μm particle size; mobile phase A: water/formic acid (1000:1); mobile phase B: acetonitrile/formic acid (1000:1); gradient typically: 0% B, 0–12 min, 0–50% B, 12–30 min, 50–100% B, 30–35 min, 100% B, 35–40 min). A single chromatographic peak (R_t_ 18.6 min, 0.2 mg) containing cnidarin 4A (**1**) as the major component along with 2 isomers was analysed by LC-MS and NMR. The crude cnidarin 4A (**1**) sample showed a [M + H]^+^ peak at *m*/*z* 517.1765 (C_20_H_29_N_4_O_12_^+^ requires 517.1783, ∆ −1.8 mmu), consistent with the molecular formula C_20_H_28_N_4_O_12_, which requires 9 degrees of unsaturation (Figure S6). Capillary ^1^H-NMR (500 MHz, Protasis CapNMR, D_2_O/H_2_O) conducted on the initial HPLC runs showed signals consistent with a polyglutamic acid (Figure S3). Further ^1^H-NMR experiments (500 MHz, 3 mm probe, D_2_O/H_2_O: gHSQC (J_CH_ = 140 Hz, relaxation delay 0.8 s); gHMBC (J_CH_ = 140 Hz, nJ_CH_ = 7 Hz, relaxation delay 0.8 s); gTOCSY (relaxation delay 1 s, spinlock mixing time 20–80 ms); gNOESY (relaxation delay 1 s, cross-relaxation delay 500 ms) confirmed signals consistent with a polyglutamic acid, while NOESY experiments showed cross peaks only between NH protons and γ-hydrogens indicating all γ-linkages between glutamic acid residues, while the degrees of unsaturation required a cyclic structure.

Cnidarin 4A (**1**) was further purified and separated from isomers by diluting venom 1:9 with 1% formic acid in water prior to injection and adjusting the initial mobile phase to 2.5% B (versus 0% B) The diluted solution was filtered through a 0.45 µm filter and fractionated by semi-preparative HPLC (Waters Atlantis dC18, 10 × 250 mm, 10 µm particles size, mobile phase A: water/formic acid (1000:1); mobile phase B: acetonitrile/formic acid (1000:1); gradient: 2.5% B, 0–7 min, 2.5–40% B, 7–24 min, 40–100% B, 24–30 min, 100% B 30–35 min). Compound **1** eluted at collected at 12–16 min.

Analyses of fractions were carried out by low resolution LC-MS using a Waters Atlantis dC18 (3 × 250 mm, 5 µm particle size) column (mobile phase A: water/formic acid (1000:1); mobile phase B: acetonitrile/formic acid (1000:1); gradient: 2.5% B, 0–7 min, 2.5–100% B, 7–27 min, 100% B, 27–35). Compound **1** was isolated as a white solid with a mass of 6.6 mg.

#### 4.4.2. Cnidarin 4A, 4B, and 4C (**1**–**3**) Isolation from *Chironex Yamaguchii*

Isolated nematocysts were extracted with distilled water by sonication with USP-400A (Shimadzu, Kyoto, Japan). The extract was centrifuged (12,000× *g*, 30 min) and the supernatant was further purified by HPLC (SCL-10A VP, LC-10ADVP, DGU-12A, Shimadzu, Kyoto, Japan) using a reversed phase column (Develosil C30, 10 × 250 mm, Nomura Chemical, Aichi, Japan) at a flow rate of 2 mL/min (mobile phase A: aqueous 50 mM HCOOH, 2 mM HCOONH_4_; mobile phase B: 5% water/95% acetonitrile with 50 mM HCOOH, 2 mM HCOONH_4_; gradient: 0–45 min, 0% B, 45–65 min, 0–5% B, 65–130 min, 5% B). Detection was by diode array, SPD-M10A VP (Shimadzu, Kyoto, Japan). The eluted peaks were collected and subject to LC-MS (positive mode). Three *m*/*z* 517 compounds were isolated and designated cnidarins 4A (**1**), 4B (**2**), and 4C (**3**) in order of elution (Figure 3).

### 4.5. Compound Characterization from Alatina alata

#### 4.5.1. Accurate Mass Determination and Routine LC-MS Analyses

Accurate mass measurements of crude cnidarin 4A, compound **1**, synthetic **1** and **2** and the synthetic intermediates were collected by LC-MS-TOF. Analyses of all samples and synthetic intermediates were performed by a low-resolution LC-MS system configured for fractionation using a 9:1 splitter with the *low flow* going to the ELSD and the *high flow* going to the PDA and MS detectors. A Waters Atlantis dC18 (3 × 250 mm, 5 µm) column was used typically with a gradient of water and acetonitrile (with 0.1% formic acid).

#### 4.5.2. NMR of Crude Cnidarin 4A (**1**)

Initial NMR was performed on crude cnidarin 4A (**1**) due to the scarcity of sample. Capillary NMR (Protasis NMRCap) was conducted with a mixture of D_2_O/H_2_O, as solvent to slow the exchange of NH protons. These experiments showed the presence of glutamate signals. Larger quantities of crude cnidarin 4A (**1**) were accumulated and analyzed in a 3 mm tube for 2D NMR [D_2_O/H_2_O: gHSQC (*J_CH_* = 140 Hz, relaxation delay 0.8 s); gHMBC (*J_CH_* = 140 Hz, *^n^J_CH_* = 7 Hz, relaxation delay 0.8 s); gTOCSY (relaxation delay 1 s, spinlock mixing time 20–80 ms); gNOESY (relaxation delay 1 s, cross-relaxation delay 500 ms)].

#### 4.5.3. Preparation of Methyl Esters of Cnidarin 4A (**1**)

Fischer esterification was performed on isolated and synthesized compound **1**. Samples of synthetic **1** (2.1 mg) and isolated **1** (2.9 mg) were dried and reconstituted in methanol (5 mL), and Amberlyst 15 Ion Exchange Resin (0.4 mg and 0.6 mg, respectively) was added to the solution. Reactions were filtered through celite (rinsing 5 × with methanol) and then a 0.45 μm PTFE filter. The solvent of the combined washings was then removed *in vacuo*. This material was further purified by flash chromatography on silica gel (20% methanol in dichloromethane). The resulting methyl esters of isolated and synthetic compound **1** were subject to ^13^C-NMR (Table 1).

#### 4.5.4. Configurational Analysis of Compound **1** Using the Advanced Marfey’s Method

The configuration of glutamic acid residues was determined for crude and isolated cnidarin 4A (**1**) by the advanced Marfey’s analysis. Residual amounts of the crude cnidarin 4A (**1**) and 22 μg of l-glutamic acid, separately, were hydrolyzed in 6 M HCl (0.2 mL, 110 °C, 18 h), dried under a stream of N_2_(g), and repeatedly reconstituted with water and redried under N_2_(g). The respective hydrolysates were reconstituted in methanol/water 1:9 and eluted from a C18 SPE column (100 mg/mL). Solvent was evaporated under a stream of N_2_(g). The hydrolysates were reconstituted in 40 µL of water and 40 µL of 1 M NaHCO_3_ solution, followed by the addition of 40 µL of l-FDLA in acetone (1% w/v solution). The mixture was heated for 3 min at 80 °C, and then acidified with 80 µL of 6 M HCl and diluted with 250 μL of acetonitrile. l-Glutamic acid standard was also reacted with dl-FDLA in the same manner. The samples were analyzed by LC-MS method below.

The configuration of cnidarin 4A (**1**) was assessed again once isolated. A portion (100 μg) of compound **1** and two 50 μg portions of l-glutamic acid were separately hydrolyzed in 6 M HCl (0.2 mL, 110 °C, 18 h), dried under a stream of N_2_(g), and repeatedly reconstituted with water and redried under N_2(g)_. The separate dried hydrolysates were reconstituted in methanol/water 1:9 and eluted from a C18 SPE column (100 mg/mL). Solvent was evaporated under a stream of N_2_(g). The dried hydrolysates were reconstituted in 40 µL of water and 50 µL of 1 M NaHCO_3_ solution, followed by the addition of 50 µL of l-FDLA in acetone (2% *w*/*v* solution). The mixture was heated for 3 min at 80 °C, and then acidified with 100 µL of 2 M HCl and diluted with 250 μL of acetonitrile. l-glutamic acid standard was also reacted with dl-FDLA.

The Marfey products for the crude and pure cnidarin 4A (**1**) hydrolysates were analyzed in comparison with standard derivatives by LC-MS on a Phenomenex Luna C18(2) (2.0 × 250 mm, 3 µm particle size) column (mobile phase A: water/formic acid (1000:1); mobile phase B: acetonitrile/formic acid (1000:1); gradient: 0–80% B, 0–30 min, 80% B, 30–35 min). Extracted ion chromatograms for corresponding derivative ions (protonated glutamic acid-FDLA monomer and dimer ions, *m*/*z* 442 and 883, respectively) were plotted and retention times compared to standards.

#### 4.5.5. Quantitation of Compound **1** in *Alatina alata* Nematocyst and Tentacles

Compound **1** in *Alatina alata* venom was quantified by LC-MS on a Waters Atlantis dC18 (3 × 250 mm, 5 μm particle size) column (mobile phase A: water/formic acid (1000:1); mobile phase B: acetonitrile/formic acid (1000:1); gradient: 2.5% B, 0–7 min, 2.5–100% B, 7–27 min, 100% B, 27–35 min), using synthetic **1** as a standard. The standard was dissolved in 0.5% aqueous formic acid and diluted in a semi-log fashion. Venom samples were prepared by diluting crude venom 1:1 with 1% aqueous formic acid to make a final concentration of 0.5% formic acid. Injection volumes were controlled by overfilling a 9.2 μL injection loop. MS/MS data was collected by fragmenting the protonated molecular ion *m*/*z* 517 and integrating the resulting MS/MS total ion chromatogram peaks were integrated using the automated selection of area under the curve in the instrument software (Qual Browser, Thermo Finnigan) with visual checked for consistency. Data for serial dilutions of the standard were collected in triplicates, and a concentration curve (Figure S8) was prepared by plotting ion counts vs. mass of the injected standard. A best-fit line was determined by linear regression. The venom sample was run in triplicate, and the compound **1** peak (9.5 min) was integrated and concentrations were calculated based on the concentration curve.

Tentacle extraction, without nematocysts, was performed with 1.4 mg of freeze-dried tentacle material that was ground and suspended in 500 µL of water, which was then diluted to 1000 µL using 1% formic acid. Samples (10 µL) were analyzed by LC-MS method above.

#### 4.5.6. ^1^H-NMR of Compound **1** and Synthetic **1**

To confirm the absolute configuration of compound **1** as the all γ-linked cyclotetraglutamic acid with a configuration of ddll, ^1^H-NMR spectral data for compound **1** was compared with that of the synthetic ddll (synthetic **1**) and dldl (synthetic **4**) isomers (Figure 2). A deuterated solution buffered with 100 mM of deuterated formic acid (pH 2.45) was used to dissolve synthetic **1** and natural **1** to ensure comparable ionization states.

### 4.6. Compound Characterization from Chironex yamaguchii

#### Marfey Analysis of Authentic Cnidarin 4C (**3**) and Synthesized llll- and dddd-cyclic tetra-γ-Glutamic Acids

For each sample, including cnidarin 4C (**3**), synthesized llll- or dddd-cyclic tetra-γ-glutamic acids (**3** and **6**, respectively), 200 μg of each was hydrolyzed by heating in a sealed vial at 120 °C for 22 h in 6 M HCl. The hydrolysate was dried with centrifugation *in vacuo*. A small portion of the acid hydrolysate was added to a 1% 1-fluoro-2,4-bis(nitrophenyl)-5-l-alanine amide (FDAA) solution in Me_2_O (10 μL) and 1 M NaHCO_3_ (20 μL). The sample was incubated for 60 min at 36 °C. The reaction mixture was neutralized with 1 M HCl (20 μL) after cooling to room temperature. The mixture was dried with centrifugation *in vacuo*. The residue was dissolved in 50 μL DMSO. The sample was analyzed by reversed phase HPLC (column: Cosmosil 5C18-AR-II 4.6 × 15 mm; flow rate: 1.2 mL/min; mobile phase: A, aqueous 0.04% trifluoroacetic acid; B, acetonitrile with 0.04% trifluoroacetic acid; gradient: 0–60 min, 10–50% B).

### 4.7. Screening of Cnidarin 4A, 4B, and 4C (***1**–**3***) in Marine Invertebrates

#### 4.7.1. Marine Species Screened for Cnidarins 4A–C (**1**–**3**)

Nine Cnidaria, one Ctenophora, one Annelida, one Mollusca, two Echinodermata, one Arthropoda, and one Chordata (17 marine animals in total) were screened for cnidarin 4A–C (**1**–**3**) by LC-MS.

Cnidaria Cubozoa:

*Chironex yamaguchii* was collected at Urasoe, Okinawa in August from 2006–2015.

*Carybdea brevipedalia* was collected at Misaki, Kanagawa in September 2010.

Cnidaria Hydrozoa:

*Physalia physalis* was collected at Banda, Chiba in August 2009.

*Millepora tenera* was collected at Aka Island, Okinawa in August 2005.

Cnidaria Scyphozoa:

*Aurelia coerulea* was collected at Tokyo-bay, Tokyo in June 2012.

*Chrysaora pacifica* was collected at Tokyo-bay, Tokyo in April 2012.

*Nemopilema nomurai* was collected at Sea of Japan, Niigata in September 2009.

Cnidaria Anthozoa:

*Phyllodiscus semoni* was collected at Itoman, Okinawa in August 2000.

*Actineria villosa* was collected at Itoman, Okinawa in August 2000.

Ctenophora Tentaculata:

*Bolinopsis mikado* was collected at Yokohama port, Kanagawa in September 2012.

Echinodermata Asteroidea:

*Certonardoa semiregularis* was collected at Zushi, Kanagawa in June 2012.

Echinodermata Echinoidea:

*Heliocidaris crassispina* was collected at Hayama, Kanagawa in June 2012.

*Echinometra mathaei* was purchased from an aquarium shop in 2012.

Annelida Polychaeta:

*Perinereis nuntia* was purchased from a fishing equipment store in 2012.

Mollusca Bivalvia:

*Mytilus galloprovincialis* was purchased from the Tsukiji fish market in 2012.

Arthropoda Malacostraca:

*Marsupenaeus japonicas* was purchased from the Tsukiji fish market in 2012.

Chordata Actinopterygii:

*Trachurus japonicus* was purchased from the Tsukiji fish market in 2012.

Whole bodies of all the samples (except *N. nomurai*) were immediately frozen after collection or purchasing and kept at −30 °C in the laboratory until analyzed. For *N. nomurai*, only the tentacles were collected and frozen. *C. yamaguchii* and *C. brevipedalia* were identified by Dr. Shin Kubota, Kyoto University. *P. physalis*, *A. coerulea*, *C. pacifica* and *N. nomurai* were identified by Dr. Haruto Ishii, Tokyo University of Marine Science and Technology. *P. semoni* and *A. villosa* were identified by Dr. Hiro’omi Uchida, Kushimoto Marine Park. *M. tenera* was identified by Dr. Yuri Latypov, Russian Academy of Science. For *C. yamaguchii*, the voucher specimens were deposited at the Okinawa Prefectural Institute of Health and Environment. For *N. nomurai*, the voucher specimens were deposited at Japan Sea National Fisheries Research Institute. For *C. brevipedalia*, *P. physalis*, *A. coerulea*, *C. pacifica*, *P. semoni*, *A. villosa* and *M. tenera*, the voucher specimens were deposited at the laboratory of aquatic ecochemistry, Tokyo University of Marine Science and Technology.

#### 4.7.2. Screening Procedure

Each sample (100 g) was homogenized in EtOH (200 mL) and filtered. Filtrate was evaporated and condensed, then liquid-liquid partition was performed between EtOAc and H_2_O. The H_2_O layer was evaporated and partition between CHCl_3_ and H_2_O. The resulting H_2_O layer was evaporated to dryness and re-dissolved with 20 mL of H_2_O and eluted from a solid phase extraction cartridge (Sep-Pak C18 Plus Long Cartridge) that was equilibrated with H_2_O. The cartridge was washed with 15 mL of H_2_O. These non-retained fractions were combined and evaporated to dryness before re-dissolving in a small volume of H_2_O and analyzing by LC-MS. The internal standard was the penta-peptide Lys-Glu-Glu-Glu-Glu, commercially synthesized by JBioS (Saitama, Japan). For sample where cnidarin 4A (**1**) was not be detected, authentic cnidarin 4B (**2**) was added to the extract and reanalyzed to confirm the ionization of cnidarin 4B (**2**) in the sample solution.

### 4.8. Synthesis of Targeted Cyclic Tetraglutamic Acids

#### 4.8.1. ddll Cyclic Tetra-γ-Glutamic Acid (Cnidarin 4A, **1**) Synthesis (See Scheme 1)

(R)-5-(Benzyloxy)-4-((R)-5-(benzyloxy)-4-((tert-butoxycarbonyl)amino)-5-oxopentanamido)-5-oxopentanoic acid (**6**)

Boc-d-glutamic acid 1-benzyl ester (**5**) was prepared by adding Boc anhydride to d-glutamic acid 1-benzyl ester and triethylamine in a solution of dichloromethane. Following work-up, EDCI (0.271 g, 1.42 mmol) was added to a solution of **5** (0.382 g, 1.13 mmol) and *N*-hydroxysucinimide (0.182 g, 1.58 mmol) in dichloromethane (5.7 mL) at room temperature. The reaction was monitored by TLC and after 18 h was diluted with dichloromethane (75 mL) and washed 3 times with saturated KH_2_PO_4_. The combined aqueous layers were back extracted with dichloromethane and the combined organic layers were washed with saturated NaCl, dried over MgSO_4_, and concentrated, providing the crude NHS ester (0.532 g, quant) as a white powder, deemed suitable for use in the subsequent reaction. To a solution of the crude NHS ester and triethylamine (0.221 mL, 0.160g, 1.59 mmol) in dichloromethane (11 mL) at room temperature, 1-benzyl d-glutamate (0.309 g, 1.30 mmol) was added. The reaction was monitored by TLC and after 21 h, diluted with dichloromethane (75 mL) and washed 3 times with saturated KH_2_PO_4_. The combined aqueous layers were back extracted with dichloromethane and the combined organic layers were washed with saturated NaCl, dried over MgSO_4_ and concentrated. The crude product was purified by flash chromatography on silica gel, (5% methanol/95% dichloromethane with 0.5% acetic acid acid) to provide **6** (0.559 g, 95% (two step yield)) as a white powder: mp 104–106 °C; R*f* = 0.31 (5% methanol/95% dichloromethane with 0.5% acetic acid); ^1^H-NMR (300 MHz, CD_3_OD) δ 7.39–7.31 (m, 10H), 5.18 (s, 2H), 5.17 (d, *J* = 3.0, 2H), 4.48 (dd, *J* = 6.0,9.0, 1H), 4.17 (dd, *J* = 3.0,9.0, 1H), 2.40–2.32 (m, 4H), 2.21–2.09 (m, 2H), 2.01–1.86 (m, 2H), 1.44 (s, 9H); ^13^C-NMR (75 MHz, CD_3_OD) δ 174.9, 173.7, 172.4, 171.7, 156.8, 136.0, 136.0, 128.3, 128.3, 128.1, 128.0, 128.0, 79.5, 66.9, 66.8, 53.7, 52.2, 31.8, 30.0, 27.7, 27.3, 26.6; IR 3333 (br), 2976 (m), 1738 (m), 1520 (m), 1455 (s) cm^−1^; TOF-MS *m*/*z* 579.2308 [M + Na]^+^ (C_29_H_36_N_2_O_9_Na^+^ requires 579.2319, ∆ −1.1 mmu). See Figures S10 and S11 for ^1^H and ^13^C-NMR spectra.

(6R,11R,16S)-6,11,16-Tris((benzyloxy)carbonyl)-2,2-dimethyl-4,9,14-trioxo-3-oxa-5,10,15-triazanonadecan-19-oic acid (**7**)

To a solution of **6** (0.575 g, 1.03 mmol) and *N*-hydroxysucinimide (0.166 g, 1.45 mmol) in dichloromethane (10 mL) at room temperature was added EDCI (0.247 g, 1.29 mmol). The reaction was monitored by TLC and after 19 h was diluted with dichloromethane (90 mL) and washed 3 × with saturated KH_2_PO_4_. The combined aqueous layers were back extracted with dichloromethane and the combined organic layers were washed with saturated NaCl, dried over MgSO_4_ and concentrated, providing the crude NHS ester (0.614 g, quant) as a white foam, deemed suitable for use in the subsequent reaction. To a solution of the crude NHS ester (all the material from the previous reaction) and triethylamine (0.201 mL, 0.146 g, 1.45 mmol) in dichloromethane (10 mL) at room temperature, 1-benzyl l-glutamate (0.282 g, 1.19 mmol) was added. The reaction was monitored by TLC and after 19 h was diluted with dichloromethane (90 mL) and washed 3 × with saturated KH_2_PO_4_. The combined aqueous layers were back extracted with dichloromethane and the combined organic layers were washed with saturated NaCl, dried over MgSO_4_ and concentrated. The crude product was purified by flash chromatography on silica gel (4% methanol/96% dichloromethane with 0.5% acetic acid) to provide **7** (0.5570 g, 71% (two step yield)) as a white foam: R*f* = 0.47 (10% methanol in dichloromethane with 0.5% acetic acid); ^1^H-NMR (300 MHz, CD_3_OD) δ 7.43–7.29 (m, 15H), 5.24–5.13 (m, 6H), 4.53–4.45 (m, 2H), 4.21 (dd, *J* = 6.0,12.0, 1H), 2.42–2.31 (m, 6H), 2.27–2.09 (m, 3H), 2.01–1.85 (m, 3H), 1.46 (s, 9H); ^13^C-NMR (75 MHz, CD_3_OD) δ 174.7, 173.6, 173.5, 172.4, 171.7, 171.6, 136.0, 136.0, 128.4, 128.3, 128.1, 128.0, 128.0, 79.6, 66.9, 66.8, 53.4, 52.3, 31.7, 31.7, 30.0, 27.7, 27.2, 26.5; IR 3321 (br), 3041 (m), 2953 (m), 2356 (s), 1737 (s), 1654 (s), 1529 (s) cm^−1^; TOF-MS *m*/*z* 798.3221 [M + Na]^+^ (C_41_H_49_N_3_O_12_Na^+^ requires 798.3214, ∆ +0.7 mmu). See Figures S12 and S13 for ^1^H and ^13^C-NMR spectra.

(6S,11S,16R,21R)-6,11,16,21-Tetrakis((benzyloxy)carbonyl)-2,2-dimethyl-4,9,14,19-tetraoxo-3-oxa-5,10,15,20-tetraazatetracosan-24-oic acid (**8**)

To a solution of **7** (0.545 g, 0.70 mmol) and *N*-hydroxysucinimide (0.056 g, 0.491 mmol) in dichloromethane (7.0 mL) at room temperature, EDCI (0.101 g, 0.526 mmol) was added. The reaction was monitored by TLC and after 15 h was diluted with dichloromethane (100 mL) and washed 3 × with saturated KH_2_PO_4_. The combined aqueous layers were back extracted with dichloromethane and the combined organic layers were washed with saturated NaCl, dried over MgSO_4_ and concentrated, providing the crude NHS ester (0.711 g, quant) as a light brown foam deemed suitable for use in the subsequent reaction. To a solution of the crude NHS ester and triethylamine (0.136 mL, 0.099 g, 0.981 mmol) in dichloromethane (7.0 mL), 1-benzyl l-glutamate (0.191 g, 0.806 mmol) was added at room temperature. The reaction was monitored by TLC and after 14 h was diluted with dichloromethane (100 mL) and washed 3 × with saturated KH_2_PO_4_. The combined aqueous layers were back extracted with dichloromethane and the combined organic layers were washed with saturated NaCl, dried over MgSO_4_ and concentrated. The crude product was purified by flash chromatography on silica gel (4–8% methanol/96–92% dichloromethane with 0.5% acetic acid) to provide **8** (0.619 g, 89% (two step yield)) as a light brown foam: R*f* = 0.29 (5% methanol/95% dichloromethane with 0.5% acetic acid); ^1^H-NMR (300 MHz, CD_3_OD) δ 7.39–7.28 (m, 20H), 5.18–5.13 (m, 8H), 4.50–4.37 (m, 3H), 4.18 (dd, *J* = 6.0,9.0, 1H), 2.41–2.27 (m, 8H), 2.23–2.07 (m, 4H), 1.99–1.82 (m, 4H), 1.43 (s, 9H); ^13^C-NMR (75 MHz, CD_3_OD) δ 174.8, 173.6, 173.50, 173.5, 172.4, 171.7, 171.6, 136.0, 136.0, 128.4, 128.35, 128.1, 128.0, 128.0, 128.0, 79.6, 66.9, 66.8, 53.5, 52.4, 52.3, 31.8, 31.7, 30.0, 27.2, 27.0, 26.5; IR 3300 (br), 3046 (m), 2958 (m), 2341 (m), 1732 (m), 1724 (m), 1648 (s) cm^−1^; TOF-MS *m*/*z* 1017.4117 [M + Na]^+^ (C_53_H_62_N_4_O_15_Na^+^ requires 1017.4109, ∆ +0.8 mmu). See Figures S14 and S15 for ^1^H and ^13^C-NMR spectra.

Tetrabenzyl(2S,7S,12R,17R)-5,10,15,20-tetraoxo-1,6,11,16-tetraazacycloicosane-2,7,12,17-tetracarboxylate (**9**)

To a solution of **8** (0.373 g, 0.375 mmol) in dichloromethane (25 mL) at 0 °C, a pre-cooled 1:1 solution of trifluoroacetic acid and dichloromethane (25 mL) were added portionwise; 5 mL was added at a time every 5 min (5×) for a total of 25 mL. The reaction was monitored by TLC by conducting “mini workups” (drying under N_2_(g) and reconstituting with dichloromethane, 3× in total). After 1 h the solvent was removed by rotary-evaporation and the crude reconstituted in dichloromethane followed again by evaporation (3× in total) to provide the free ammonium trifluoroacetate as a brown foam. To a solution of the crude product, all material from previous reaction, and triethylamine (0.157 mL, 0.114 g, 1.12 mmol) in dichloromethane (375 mL) at room temperature, EDCI (0.216 g, 1.12 mmol) was added. The reaction was monitored by TLC and after 9 h was diluted with dichloromethane (100 mL) and washed 3 × with saturated KH_2_PO_4_. The combined aqueous layers were back extracted with dichloromethane and the combined organic layers were washed with saturated NaCl, dried over MgSO_4_ and concentrated. The crude product was purified by recrystallization according to the following procedure. The crude sample was dissolved in warm dichloromethane, filtered through cotton in a glass funnel followed by the addition of a small volume of hexane. The solution was placed in a fume hood overnight and then in a freezer for 4 days providing small crystals. The suspension was centrifuged, and the supernatant removed followed by 2 hexane-wash/centrifugation cycles to provide **9** (123.5 mg, 37% two step yield) as a white powder: mp 244 - 246 °C; ^1^H-NMR (300 MHz, CDCl_3_) δ 7.38–7.27 (m, 20H), 7.095 (d, *J*=3.0, 2H), 6.94 (d, *J*=6.0, 2H), 5.17 (qd, *J* = 6.0,13.5, 8H), 4.60–4.49 (m, 2H), 4.40–4.31 (m, 2H), 2.50–1.98 (m, 16H); ^13^C-NMR (75 MHz, CDCl_3_) δ 173.6, 173.3 171.6, 171.4, 135.8, 135.7, 128.8, 128.6, 128.5, 128.2, 128.0, 67.4, 67.2, 54.0, 53.9, 32.1, 31.2, 25.3, 24.3; IR 3290 (br), 3051 (m), 2932 (m), 1732 (s), 1638 (m), 1534 (s) cm^−1^; TOF-MS *m*/*z* 899.3425 [M + Na]^+^ (C_48_H_52_N_4_O_12_Na^+^ requires 899.3479, ∆ −0.54 mmu). See Figures S16 and S17 for ^1^H and ^13^C-NMR spectra.

2.1,5.4-Anhydro(γ-d-glutamyl-γ-d-glutamyl-γ-l-glutamyl-l-glutamic acid), cnidarin 4A (**1**)

Above a solution of **9** (20.2 mg, 0.023 mmol) and 10% Pd/C (~2 mg) in ethanol (20 mL) at room temperature, hydrogen gas was maintained at ambient pressure for 24 h. The reaction was then filtered through a PTFE filter followed by ethanol washing (5 × 1 mL), methanol washing (5 × 1 mL) then water (2 × 1.5 mL). The crude product was purified by HPLC using a Waters Atlantis dC18 (10 × 250 mm, 10 μm particle size) column (mobile phase A: water/formic acid (1000:1); mobile phase B: acetonitrile/formic acid (1000:1); gradient: 2.5% B, 0–7 min, 2.5–100% B, 7–27 min, 100% B, 27–35 min) to provide cnidarin 4A, **1** (9.7 mg, 79%) as a white solid: decomp 279–285 °C; ^1^H-NMR (500 MHz, D_2_O) δ 4.33 (dd, *J*=5.0,12.0, 2H), 4.255 (dd, *J*=5.0,10.0, 2H), 2.47–2.30 (m, 10H), 2.15–2.05 (m, 2H), 2.03–1.94 (m, 2H), 1.93–1.84 (m, 2H); ^13^C-NMR (125 MHz, D_2_O) δ 178.4, 178.2, 177.9, 177.5, 55.4, 54.3, 34.5, 33.8, 29.0, 28.6; IR 3286 (br), 2560 (br), 1724 (m), 1538 (m), 1415 (br) cm^−1^; TOF-MS *m*/*z* 539.1578 [M + Na]^+^ (C_20_H_28_N_4_O_12_Na^+^ requires 539.1601, ∆ −2.3 mmu). See Figures S18 and S19 for ^1^H and ^13^C-NMR spectra.

#### 4.8.2. llll Cyclic Tetraglutamic Acid (Cnidarin 4C, **3**) Synthesis (See Scheme 2)

5-Benzyl 1-(tert-butyl)((S)-5-(tert-butoxy)-4-((tert-butoxycarbonyl)amino)-5-oxopentanoyl)-l-glutamate (**12**)

To a solution of **10** (1.09 g, 3.61 mmol) in dichloromethane (15 mL), DMAP (220 mg, 1.8 mmol), EDCI (1.38 g, 7.22 mmol) and **11** (1.06 g, 3.06 mmol) were added, and the reaction mixture was stirred at room temperature for 2 h. H_2_O was added to the reaction mixture and the organic layer was extracted with dichloromethane. The extracts were dried over MgSO_4_, filtered, and concentrated *in vacuo*. The residue was purified by column chromatography to give **12** (1.64 g, 2.83 mmol) in 78% yield.

5-Benzyl 1-(tert-butyl) ((S)-4-amino-5-(tert-butoxy)-5-oxopentanoyl)-l-glutamate (**13**)

To a solution of **12** (0.9 g, 1.55 mmol) in CH_3_CN/H_2_O (50:1), BiCl_3_ was added portionwise (977 mg, 3.1 mmol), to selectively deprotect the Boc group [40], NaHCO_3_ was added and the reaction mixture was filtered through Celite pad and and filtrates were concentrated *in vacuo* to give **13** (660 mg, 1.38 mmol) in 88% yield.

(S)-5-(tert-Butoxy)-4-((S)-5-(tert-butoxy)-4-((tert-butoxycarbonyl)amino)-5-oxopentanamido)-5-oxopentanoic acid (**14**)

Again, to the solution of **12** (0.7 g, 1.2 mmol), Pd/C (70 mg) was added and the reaction mixture was stirred at room temperature under hydrogen gas at ambient pressure. After 20 min, the reaction mixture was filtered through a Celite pad, and filtrates were concentrated *in vacuo* to give **14** (550 g, 1.12 mmol) in 93% yield.

23-Benzyl 11,16,21,6-tetra-tert-butyl (6R,11R,16R,21R)-2,2-dimethyl-4,9,14,19-tetraoxo-3-oxa-5,10,15,20-tetraazatricosane-6,11,16,21,23-pentacarboxylate (**15**)

To a solution of **14** (660 mg, 1.38 mmol) in dichloromethane (15 mL), EDCI (528 mg, 2.76 mmol), DMAP (84.3 mg, 0.69 mmol), and the amine **13** (673 mg, 1.38 mmol) were added, and the mixture was stirred at room temperature for 2 h. To the reaction mixture was added H_2_O, and the organic layer was extracted by dichloromethane. The extracts were dried over MgSO_4_, filtered, and concentrated *in vacuo*. The residue was purified by column chromatography to give **15** (1.0 g, 1.05 mmol) in 79% yield.

2.1,5.4-Anhydro(γ-l-glutamyl-γ-l-glutamyl-γ-l-glutamyl-l-glutamic acid), cnidarin 4C (**3**)

To a solution of **15** (1.0 g, 1.05 mmol) in CH_3_CN-H_2_O (50:1), BiCl_3_ was added portionwise (662 mg, 2.1 mmol) to selectively deprotect the Boc group.^1^ NaHCO_3_ was added and the reaction mixture was filtered through a Celite pad, and filtrates were concentrated *in vacuo*. The concentrates further were purified by column chromatography to give the linear llll-tetraglutamic acid (420 mg, 0.49 mmol) in 46% yield. [α]_D_^25^ +7.34° (*c* 1.88, CHCl_3_); ^1^H-NMR (400 MHz, CDCl_3_) δ 7.32 (s, 5H), 7.07 (s, 1H), 7.05 (s, 1H), 5.10 (s, 2H), 4.46–4.43 (m, 3H), 3.71 (m, 1H), 2.53–2.39 (m, 4H), 2.35–2.25 (m, 4H), 2.24–2.11 (m, 4H), 2.02–1.76 (m, 4H), 1.48–1.42 (m, 36H); ^13^C-NMR (100 MHz, CDCl_3_) δ 172.6, 172.5, 172.2, 172.0, 171.2, 171.0, 135.7, 128.4, 128.1, 82.1, 66.3, 53.7, 52.2, 52.0, 32.2, 31.8, 30.3, 28.8, 28.5, 28.3, 27.8, 27.4 ppm; HRMS (ESI, [M + Na]^+^) *m*/*z* 871.4697 (calcd for C_43_H_68_N_4_O_13_Na^+^ 871.4680, ∆ +1.7 mmu). See Figures S30 and S31 for ^1^H and ^13^C-NMR spectra.

To the linear llll-glutamic acid (126 mg, 0.14 mmol) in THF, Pd/C (12 mg) was added and hydrogen gas was maintained at ambient pressure to remove the benzyl group. The reaction mixture was filtrate through Celite pad and concentrated *in vacuo.* The concentrates (166 mg, 0.21 mmol) were subjected to macrocylization in the presence of 2-methyl-6-nitrobenzoic anhydride (MNBA, 108 mg, 0.315 mmol), DMAP (2.56 g, 0.021 mmol) and Et_3_N (175 mL, 1.26 mmol) to give the cyclic llll-glutamic acid and finally the Boc group deprotected by TFA to give **3** (48.6 mg, 0.094 mmol) in 67% yield in three steps. [α]_D_^25^ −28.4 (*c* 0.83, methanol); ^1^H-NMR (400 MHz, D_2_O) δ 4.44–4.28 (m, 4H), 2.55–2.32 (m, 8H), 2.28, 2.14 (m, 4H), 2.05–1.87 (m, 4H); ^13^C-NMR (100 MHz, D_2_O) δ 175.7, 175.6, 175.5, 175.4, 53.6, 52.5, 52.3, 31.7, 26.6 ppm; HRMS (ESI, [M + Na]^+^) *m*/*z* 539.1589 (calcd for C_20_H_28_N_4_O_12_Na^+^ 539.1601, ∆ −1.2 mmu). See Figures S32 and S33 for ^1^H and ^13^C-NMR spectra.

### 4.9. Bioassays

#### 4.9.1. Cytotoxicity Assay

Sample preparation: Cnidarin 4A (**1**) (2.1,5.4-anhydro(γ-d-glutamyl-γ-d-glutamyl-γ-l-glutamyl-l-glutamate) and *iso*-cnidarin 4A (**4**) (2.1,5.4-anhydro(γ-d-glutamyl-γ-l-glutamyl-γ-d-glutamyl-l-glutamate) (MW 516) samples were dissolved in 5% bovine serum (FBS) (Mediatech) supplemented Dulbecco’s Modified Eagle Medium (DMEM) (Sigma Aldrich) media, with vortexing and sonicating to allow complete solvation, and sterile filtered. Doxorubicin dissolved in 5% FBS supplemented DMEM media was used as a toxicity standard. Compounds were half-log serial diluted.

Cell culture: HEK-293 cells were cultured in DMEM supplemented with 10% FBS and 1% penicillin-streptomycin (Mediatech) and maintained at 37 °C, 90% humidity, and 5% CO_2_ atmosphere.

Bioassay: Cells were plated in a 96-well plate at 10,000 cells/well in DMEM supplemented with 5% FBS and 1% penicillin-streptomycin. Cells were incubated for 24 h prior to compound addition to allow cells to grow to confluency. Next 100 µL of (2×) compound or control was added to the wells, and plates were incubated (37 °C, 90% humidity, and 5% CO_2_) for 48 h. Cells were then fixed with 50% cold trichloroacetic acid (TCA), added directly to the wells, and allowed to sit for 1 h at 4 °C. Plates were washed with tap water to remove media and TCA, and dried. 0.4% sulforhodamine B in 1% acetic acid was added to each well and allowed to sit for 30 min at room temperature. Plates were then washed 5–6 times with 1% acetic acid and dried. Dye was solubilized with 10 mM Tris base (pH = 10.5) and allowed to sit for 30 min until dye was completely solubilized. Plates were then read for absorbance on the Flexstation 3 (Molecular Devices) at 500 nm and a background wavelength of 690 nm.

Background absorbance was subtracted from all experiment wells. Growth inhibition/lethality was calculated using four parameters: (1) T_z_: absorbance measurement of cells fixed at “time zero”, the same time test compounds were added to other wells, and represented 0% growth. (2) T_c_: absorbance measurement of control cells grown in media only, represented 100% growth. (3) No Cells: absorbance measurement of background control (wells with no cells), and represented 100% lethality. (4) T_i_: absorbance measurement of cells grown in test compound. Percent growth inhibition was calculated ([(T_i_ − T_z_)/(T_c_ − T_z_)] × 100) if absorbance of test compound (T_i_) is greater than absorbance of cells fixed at time = 0 (T_z_). Compounds **1** and **2** showed not notable reduction of cell growth or survival at any concentration tested (see Figure S9).

#### 4.9.2. Hemolysis Assay

A 6% red blood cell (RBC) solution in 140 mM NaCl was prepared from human whole blood. Compound **1** was dissolved in 140 mM NaCl at 2 concentrations, 98 and 484 μM, and a 10% TritonX-100 (Fisher Scientific) solution was prepared in 110 mM NaCl. In a 96-well plate, 100 μL of the RBC solution was combined with 100 μL of vehicle (140 mM NaCl) or diluted stock solutions of compound **1** or TritonX-100 (in 140 mM NaCl) to give final compound **1** concentrations of 24 and 121 μM and a 1% TritonX-100 concentration. Each condition was tested in triplicate. The plate was incubated at 37 °C for 1 h before adding 70 μL 140 mM NaCl and centrifuging at 750× *g* for 10 min at 4 °C (Marathon 2100R Centrifuge, Fisher Scientific). A 100 μL aliquot of each supernatant was then transferred to a flat bottom 96-well plate, and the absorbance was read at 405 nm on an Ultramark EX Microplate Imaging System (Bio-Rad) to quantitate the hemoglobin concentration as a measure of the extent of hemolysis. The hemoglobin concentration was calculated from its molar absorptivity and the data was normalized to the signal for 1% TritonX-100 (positive control). Student t-test showed compound **1** caused no significant hemolysis compared to negative control (saline) at either concentration. See Table S2.

## 5. Conclusions

Three new cyclic γ-linked tetraglutamic acids, cnidarins 4A (**1**), 4B (**2**), and 4C (**3**), were isolated from venom extracts of the jellyfish *Alatina alata* and *Chironex yamaguchii*. The full configurational assignments were confirmed by syntheses. The most abundant of these compounds, cnidarin 4A (**1**), showed no mammalian cell toxicity or hemolytic activity. These compounds were detected in all nine Cnidarians tested, but not in the other nine non-Cnidarians species examined (one Ctenophora, one Annelida, one Mollusca, two Echinodermata, one Arthropoda, and one Chordata). Although a limited number of invertebrate species were screened, the current results may be an indication that these compounds are specific to Cnidaria. In light of the fact that no hemolytic or toxic effects were detected for the most abundant of the peptides, the role of cnidarins 4A (**1**), 4B (**2**), and 4C (**3**) in the Cnidarians’ nematocyst is an intriguing research topic that needs to be resolved.

## Figures and Tables

**Table 1:** ^13^C-NMR of tetramethyl esters of **1** isolated from *A. alata* venom and synthesized.

δ_C_	
Isolated 1 Tetramethyl Ester	Synthetic 1 Tetramethyl Ester
25.83	25.81
26.63	26.61
30.73	30.69
31.42	31.38
50.09	50.07
51.38	51.36
51.94	51.91
51.98	51.96
170.69	170.65
172.19	172.17
172.56	172.55
172.82	172.81

**Table 2:** ^1^H-NMR (800 MHz) and ^13^C-NMR (200 MHz) for cnidarin 4A (**1**) in CD_3_OD.

Position	^13^C-NMR (ppm)	^1^H-NMR (ppm)	Mult.	J (Hz)
Glu-1 ^a^				
COOH	177.4	-		
α	54.5	4.37	dd	2.4, 6.6
β	28.7	Hα 1.89 Hβ 2.35	m m	
γ	33.8	2H 2.41	m	
C=O	174.8			
Glu-2 ^a^				
COOH	177.8	-		
α	55.3	4.27	dd	2.4, 6.6
β	29.6	Hα 2.01 Hβ 2.13	m m	
γ	33.0	Hα 2.31 Hβ 2.38	m m	
C=O	175.4			

^a^ The positions of Glu-1 and Glu-2 were not determined.

**Table 3:** ^1^H-NMR (800 MHz) and ^13^C-NMR (200 MHz) for cnidarin 4B (**2**) in CD_3_OD.

Position	^13^C-NMR (ppm)	^1^H-NMR (ppm)	Mult.	J (Hz)
Glu-1 ^a^				
COOH	176.42 ^b^	-		
α	54.15	4.37	m	
β	28.71	Hα 1.94 Hβ 2.35	m m	
γ	33.8	Hα 1.94 Hβ 2.35	m m	
C=O	174.8			
Glu-2 ^a^				
COOH	176.43 ^b^	-		
α	54.38	4.34	m	
β	28.27	Hα 2.02 Hβ 2.22	m m	
γ	32.87	Hα 2.36 Hβ 2.46	m m	
C=O	175.3			
Glu-3 ^a^				
COOH	176.65 ^c^	-		
α	54.8	4.32	m	
β	29.11	Hα 2.06 Hβ 2.16	m m	
γ	33.71	Hα 2.36 Hα 2.41	m m	
C=O	175.07			
Glu-4^a^				
COOH	176.66 ^c^	-		
α	54.38	4.33	m	
β	28.81	Hα 2.02 Hβ 2.16	m m	
γ	33.81	2H 2.41	m	
C=O	175.24			

^a^ The positions of Glu-1, Glu-2, Glu-3, and Glu-4 were not determined. ^b,c^ Signals assigned may be interchanged.

**Table 4:** ^1^H-NMR (800 MHz) and ^13^C-NMR (200 MHz) for cnidarin 4C (**3**) in CD_3_OD.

Position	^13^C-NMR (ppm)	^1^H-NMR (ppm)	Mult.	J (Hz)
COOH	175.6	-		
α	53.3	4.41	dd	1.4, 6.5
β	28.2	Hα 1.99 Hβ 2.31	m m	
γ	32.8	2H 2.41	m	
C=O	175.0			

## Supplementary Materials

The following are available online at https://www.mdpi.com/1420-3049/25/4/883/s1: Methods and results for synthesis of compounds **2** and **4**, chromatography and chemical analyses of compound **1**, cell toxicity assays of compounds **1** and **4**, and NMR spectra of synthetic intermediates for compounds **1**–**4**.

## References

[1] Bentlage B., Cartwright P., Yanagihara A.A., Lewis C., Richards G.S., Collins A.G. (2009). Evolution of box jellyfish (Cnidaria: Cubozoa), a group of highly toxic invertebrates. Proc. R. Soc. B Biol. Sci..

[2] Fenner P.J., Harrison S.L. (2000). Irukandji and *Chironex fleckeri* jellyfish envenomation in tropical Australia. Wilderness Environ. Med..

[3] Ramasamy S., Isbister G.K., Seymour J.E., Hodgson W.C. (2004). The in vivo cardiovascular effects of box jellyfish *Chironex fleckeri* venom in rats: Efficacy of pre-treatment with antivenom, verapamil and magnesium sulphate. Toxicon.

[4] Currie B.J. (2003). Marine antivenoms. J. Toxicol. Clin. Toxicol..

[5] Brinkman D., Burnell J. (2007). Identification, cloning and sequencing of two major venom proteins from the box jellyfish, *Chironex fleckeri*. Toxicon.

[6] Tibballs J. (2006). Australian venomous jellyfish, envenomation syndromes, toxins and therapy. Toxicon Off. J. Int. Soc. Toxinol..

[7] Carrette T.J., Underwood A.H., Seymour J.E. (2012). Irukandji syndrome: A widely misunderstood and poorly researched tropical marine envenoming. Diving Hyperb. Med..

[8] Tibballs J., Li R., Tibballs H.A., Gershwin L.-A., Winkel K.D. (2012). Australian carybdeid jellyfish causing “Irukandji syndrome”. Toxicon.

[9] Yanagihara A.A., Wilcox C., Smith J., Surrett G.W. (2016). Cubozoan Envenomations: Clinical Features, Pathophysiology and Management. The Cnidaria, Past, Present and Future.

[10] Lewis Ames C., Bentlage B. (2009). Clarifying the identity of the Japanese Habu-Kurage, C*hironex yamaguchii*, sp. nov. (Cnidaria: Cubozoa: Chirodropida). Zootaxa.

[11] Horiike T., Nagai H., Kitani S. (2015). Identification of allergens in the box jellyfish *Chironex yamaguchii* that cause sting dermatitis. Int. Arch. Allergy Immunol..

[12] Lewis C., Bentlage B., Yanagihara A., Gillan W., Blerk J.V., Keil D.P., Bely A.E., Collins A.G. (2013). Redescription of *Alatina alata* (Reynaud, 1830) (Cnidaria: Cubozoa) from Bonaire, Dutch Caribbean. Zootaxa.

[13] Nagai H., Takuwa K., Nakao M., Sakamoto B., Crow G.L., Nakajima T. (2000). Isolation and characterization of a novel protein toxin from the Hawaiian box jellyfish (sea wasp) *Carybdea alata*. Biochem. Biophys. Res. Commun..

[14] Yoshimoto C.M., Yanagihara A.A. (2002). Cnidarian (coelenterate) envenomations in Hawai’i improve following heat application. Trans. R. Soc. Trop. Med. Hyg..

[15] Anderluh G., Sepčić K., Turk T., Maček P. (2011). Cytolytic proteins from cnidarians-an overview. Acta Chim. Slov..

[16] Mariottini G.L., Pane L. (2013). Cytotoxic and cytolytic cnidarian venoms. A review on health implications and possible therapeutic applications. Toxins.

[17] Brinkman D.L., Burnell J.N. (2009). Biochemical and molecular characterisation of cubozoan protein toxins. Toxicon.

[18] Chung J.J., Ratnapala L.A., Cooke I.M., Yanagihara A.A. (2001). Partial purification and characterization of a hemolysin (CAH1) from Hawaiian box jellyfish (*Carybdea alata*) venom. Toxicon.

[19] Yanagihara A.A., Shohet R.V. (2012). Cubozoan venom-induced cardiovascular collapse is caused by hyperkalemia and prevented by zinc gluconate in mice. PLoS ONE.

[20] Brinkman D.L., Jia X., Potriquet J., Kumar D., Dash D., Kvaskoff D., Mulvenna J. (2015). Transcriptome and venom proteome of the box jellyfish *Chironex fleckeri*. BMC Genom..

[21] Jouiaei M., Casewell N., Yanagihara A., Nouwens A., Cribb B., Whitehead D., Jackson T., Ali S., Wagstaff S., Koludarov I. (2015). Firing the sting: Chemically induced discharge of cnidae reveals novel proteins and peptides from box jellyfish (*Chironex fleckeri*) venom. Toxins.

[22] Jaimes-Becerra A., Chung R., Morandini A.C., Weston A.J., Padilla G., Gacesa R., Ward M., Long P.F., Marques A.C. (2017). Comparative proteomics reveals recruitment patterns of some protein families in the venoms of Cnidaria. Toxicon.

[23] Fenner P.J., Hadok J.C. (2002). Fatal envenomation by jellyfish causing Irukandji syndrome. Med. J. Aust..

[24] Winkel K.D., Tibballs J., Molenaar P., Lambert G., Coles P., Ross-Smith M., Wiltshire C., Fenner P.J., Gershwin L.-A., Hawdon G.M. (2005). Cardiovascular actions of the venom from the Irukandji (*Carukia Barnesi*) jellyfish: Effects in human, rat and guinea-pig tissues in vitro and in pigs in vitro. Clin. Exp. Pharmacol. Physiol..

[25] Nagai H. (2003). Recent progress in jellyfish toxin study. J. Health Sci..

[26] Ramasamy S., Isbister G.K., Seymour J.E., Hodgson W.C. (2005). Pharmacologically distinct cardiovascular effects of box jellyfish (*Chironex fleckeri*) venom and a tentacle-only extract in rats. Toxicol. Lett..

[27] Bailey P.M., Little M., Jelinek G.A., Wilce J.A. (2003). Jellyfish envenoming syndromes: Unknown toxic mechanisms and unproven therapies. Med. J. Aust..

[28] Lau M.-T., Manion J., Littleboy J.B., Oyston L., Khuong T.M., Wang Q.-P., Nguyen D.T., Hesselson D., Seymour J.E., Neely G.G. (2019). Molecular dissection of box jellyfish venom cytotoxicity highlights an effective venom antidote. Nat. Commun..

[29] Little M., Pereira P., Carrette T., Seymour J. (2006). Jellyfish responsible for Irukandji syndrome. QJM.

[30] Rocha J., Peixe L., Gomes N., Calado R. (2011). Cnidarians as a source of new marine bioactive compounds—An overview of the last decade and future steps for bioprospecting. Mar. Drugs.

[31] (2019). Dictionary of Natural Products.

[32] Lindquist N. (2002). Tridentatols D−H, nematocyst metabolites and precursors of the activated chemical defense in the marine hydroid *Tridentata marginata* (Kirchenpauer 1864). J. Nat. Prod..

[33] Munekata E., Ishiyama H., Osakada F., Izumi K. (1980). Synthetic cyclopeptides with some neuroactive properties. Pept. Chem..

[34] Hollosi M., Katar M. (1972). Synthesis of cyclo-γ-oligoglutamic acid derivatives. Acta Chim. Acad. Sci. Hung..

[35] Weber J. (1990). Poly(gamma-glutamic acid)s are the major constituents of nematocysts in *Hydra* (Hydrozoa, Cnidaria). J. Biol. Chem..

[36] Weber J. (1991). A novel kind of polyanions as principal components of cnidarian nematocysts. Comp. Biochem. Physiol. A Physiol..

[37] Szczepanek S., Cikala M., David C.N. (2002). Poly-gamma-glutamate synthesis during formation of nematocyst capsules in *Hydra*. J. Cell Sci..

[38] Berking S., Herrmann K. (2006). Formation and discharge of nematocysts is controlled by a proton gradient across the cyst membrane. Helgol. Mar. Res..

[39] Park S., Piriatinskiy G., Zeevi D., Ben-David J., Yossifon G., Shavit U., Lotan T. (2017). The nematocyst’s sting is driven by the tubule moving front. J. R. Ssoc. Interface.

[40] Navath R.S., Pabbisetty K.B., Hu L. (2006). Chemoselective deprotection of *N*-Boc group in amino acids and peptides by bismuth(III) trichloride. Tetrahedron Lett..

