# Supplementary material for: Isolation, Structure Determination, and Synthesis of Cyclic Tetraglutamic Acids from Box Jellyfish Species Alatina alata and Chironex yamaguchii
Source: Molecules. 2020 Feb 17;25(4):883. doi: 10.3390/molecules25040883 (PMC7070617; doi:10.3390/molecules25040883)
Supplement: Supplementary file 1 [file molecules-25-00883-s001.pdf]

molecules

Supplemental Information

Isolation, Structure Determination, and Synthesis of Cyclic Tetraglutamic Acids from the Box Jellyfish Species *Alatina alata* and *Chironex yamaguchii*

Justin Reinicke [1,2], Ryuju Kitatani [3], Shadi Sedghi Masoud [4], Kelly Kawabata Galbraith [1,5], Wesley Yoshida [6], Ayako Igarashi [3], Kazuo Nagasawa [4], Gideon Berger [1,*], Angel Yanagihara [7,*], Hiroshi Nagai [3,*] and F. David Horgen [1,*]

[1] Department of Natural Sciences, Hawaii Pacific University, Kaneohe, HI 96744, USA; reinicke@hawaii.edu (J.R.); kgalbraith2468@gmail.com (K.K.G.)
[2] Daniel K. Inouye College of Pharmacy, University of Hawaii at Hilo, Hilo, HI 96720, USA
[3] Department of Marine Sciences, Tokyo University of Marine Science and Technology, Tokyo 108-8477, Japan; ryujukitatani@gmail.com (R.K.); igarashi.b.ayako.t@gmail.com (A.I.)
[4] Department of Biotechnology and Life Science, Tokyo University of Agriculture and Technology, Tokyo 184-8588, Japan; shadi@m2.tuat.ac.jp (S.S.M.); knaga@cc.tuat.ac.jp (K.N.)
[5] Present address: German Center for Neurodegenerative Diseases (DZNE), Sigmund-Freud-Str. 27, 53127 Bonn, Germany
[6] Department of Chemistry, University of Hawaii at Manoa, Honolulu, HI 98622, USA; wyoshida@hawaii.edu
[7] Békésy Laboratory of Neurobiology, Pacific Biosciences Research Center, School of Ocean and Earth Science and Technology, and Department of Tropical Medicine, John A. Burns School of Medicine, University of Hawaii at Manoa, Honolulu HI 96822, USA
* Correspondence: gberger@hpu.edu (G.B.); ayanagih@hawaii.edu (A.Y.); nagai@kaiyodai.ac.jp (H.N.); dhorgen@hpu.edu (F.D.H); Tel.: +01-808-236-3551 (G.B.); +01-808- 956-8328 (A.Y.); +81-3-5463-0454 (H.N.); +01-808-236-5864 (F.D.H.)

Contents

Section S1. Synthesis of DLLL cyclic tetraglutamic acid cnidarin 4B (2) 2
Section S2. Synthesis of DLDL cyclic tetraglutamic acid *iso*-cnidarin 4A (4) 4
Section S3. Chromatography and chemical analyses of cnidarin 4A (1) 8
Section S4. Cell toxicity and hemolytic activity of cnidarin 4A (1). 14
Section S5. NMR spectra of synthetic cnidarin 4A (1) and intermediates 8-12. 15
Section S6. NMR spectra of synthetic *iso*-cnidarin 4A (4) and intermediates S5-S8...... 20
Section S7. NMR spectra of synthetic cnidarin 4C (3) and intermediates. 25
Section S8. NMR spectra of synthetic cnidarin 4B (2) and intermediates. 27

Section S1. Synthesis of DLLL cyclic tetraglutamic acid cnidarin 4B (2)

Scheme S1. Preparation of cnidarin 4B (2).

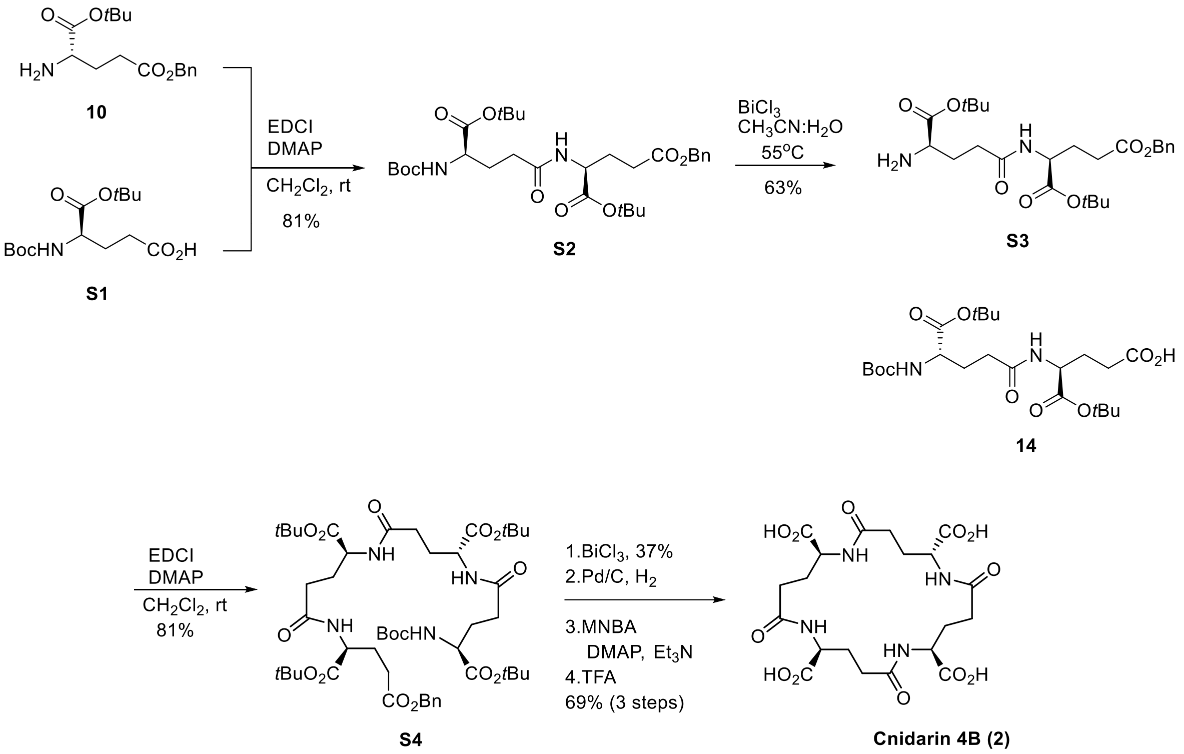

5-Benzyl 1-(tert-butyl) ((R)-5-(tert-butoxy)-4-((tert-butoxycarbonyl)amino)-5-oxopentanoyl)-L-glutamate (**S2**)

To a solution of **S1** (1.03 g, 3.4 mmol) in dichloromethane (15 mL), DMAP (200 mg, 1.7 mmol), EDCI (1.3 g, 6.8 mmol) and **10** (1 g, 3.4 mmol) were added, and the reaction mixture was stirred at room temperature for 2 hr. H_2O was added, and the organic layer was extracted with dichloromethane. The extract was dried over $MgSO_4$, filtered, and concentrated *in vacuo*. The residue was purified by column chromatography to give **S2** (1.6 g, 2.76 mmol) in 81% yield.

5-Benzyl 1-(tert-butyl) ((R)-4-amino-5-(tert-butoxy)-5-oxopentanoyl)-L-glutamate (**S3**)

To a solution of **S2** (0.9 g, 1.55 mmol) in CH_3CN-H_2O (50:1), $BiCl_3$ was added portionwise (974.4 mg, 3.1 mmol) to selectively deprotect the Boc group. $NaHCO_3$ was added, and the reaction mixture was filtered through a Celite pad to give **S3** (0.83 g, 1.73 mmol) in 63% yield.

23-Benzyl 11,16,21,6-tetra-tert-butyl (6R,11S,16R,21R)-2,2-dimethyl-4,9,14,19-tetraoxo-3-oxa-5,10,15,20-tetraazatricosane-6,11,16,21,23-pentacarboxylate (**S4**)

To a solution of amine **14** (732 mg, 1.53 mmol) in dichloromethane, EDCI (585 mg, 3.06 mmol), DMAP (93 mg, 0.765 mmol) and **S3** (749 mg, 1.53 mmol) were added, and the mixture was stirred at room temperature for 2 hr. H_2O was added to the reaction mixture, and the organic layer was extracted with dichloromethane. The extracts were dried over $MgSO_4$, filtered, and concentrated *in vacuo*. The residue was purified by column chromatography to give **S4** (1.18 g, 1.24 mmol) in 81% yield.

2.1,5.4-Anhydro(γ-L-glutamyl-γ-L-glutamyl-γ-D-glutamyl-L-glutamic acid) (cnidarin 4B, **2**)

To a solution of **S4** (1.18 g, 1.24 mmol) in CH_3CN-H_2O (50:1), $BiCl_3$ was added portionwise (782 mg, 2.48 mmol) to selectively deprotect the Boc group. $NaHCO_3$ was added and the reaction mixture was filtered through a Celite pad, and filtrates were concentrated *in vacuo*. The reaction product was further purified by column chromatography to gave the corresponding linear LLDL-tetraglutamate (390 mg, 0.459 mmol) in 37% yield: $[\alpha]_D^{25}$ +2.9° (*c* 0.68, $CHCl_3$); ^{1}H NMR (400 MHz, $CDCl_3$) δ 7.34 (S, 5H), 7.15 (s, 1H), 7.13 (s, 1H), 5.10 (s, 2H), 4.51-4.38 (m, 3H), 3.71-3.65 (m, 1H), 2.61-2.41 (m, 4H), 2.4-2.24 (m, 4H), 2.23-2.11 (m, 4H), 2.05-1.89 (m, 4H), 1.51-1.39 (m, 36H); ^{13}C NMR (75 MHz, $CDCl_3$) δ 172.9, 172.5, 172.3, 172.2, 171.1, 136.6, 128.5, 128.2, 82.2, 82.0, 66.4, 52.4, 52.1, 32.4, 30.4, 28.2, 27.9, 27.2 ppm; HRMS-ESI *m/z* 871.4708 $[M+Na]^+$ (calcd for $C_{43}H_{68}N_4O_{13}Na^+$ *m/z* 871.4680, Δ +2.8 mmu. See Figures S34 and S35 for ^{1}H and ^{13}C NMR spectra. To this product (192 mg, 0.22 mmol) in THF, Pd/C (20 mg) was added, and H_2 was exposed to the mixture to remove the benzyl group. The reaction mixture was filtered through a Celite pad and concentrated *in vacuo*. The product concentrate (161 mg, 0.212 mmol) was subjected to macrocylization in the presence of MNBA (109 mg, 0.318 mmol), DMAP (2.6 mg, 0.0212 mmol), and Et_3N (176. 3 mL, 1.272 mmol) to give the protected cyclic LLDL-glutamic acid. Finally, the Boc group was deprotected using TFA to give cnidarin 4B (**2**) (78 mg, 0.152 mmol) in 69% yield in three steps. $[\alpha]_D^{25}$ -15° (*c* 0.94, MeOH); ^{1}H NMR (400 MHz, D_2O) δ 4.42-4.28 (m, 4H), 2.52-2.32 (m, 8H), 2.29-2.11 (m, 4H), 2.09-1.89 (m, 4H); ^{13}C NMR (75 MHz, D_2O) δ 175.9, 175.7, 175.6, 175.4, 53.6, 53.1, 52.7, 52.1, 32.7, 32.3, 31.8, 31.2, 26.7, 26.5, 26.2 ppm; HRMS-ESI *m/z* 539.1542 $[M+Na]^+$ (calcd for $C_{20}H_{28}N_4O_{12}Na^+$ *m/z* 539.1601, Δ -5.9 mmu). See Figures S36 and S37 for ^{1}H and ^{13}C NMR spectra.

Section S2. Synthesis of DLDL cyclic tetraglutamic acid *iso*-cnidarin 4A (4)

Scheme S2. Preparation *iso*-cnidarin 4A (4).

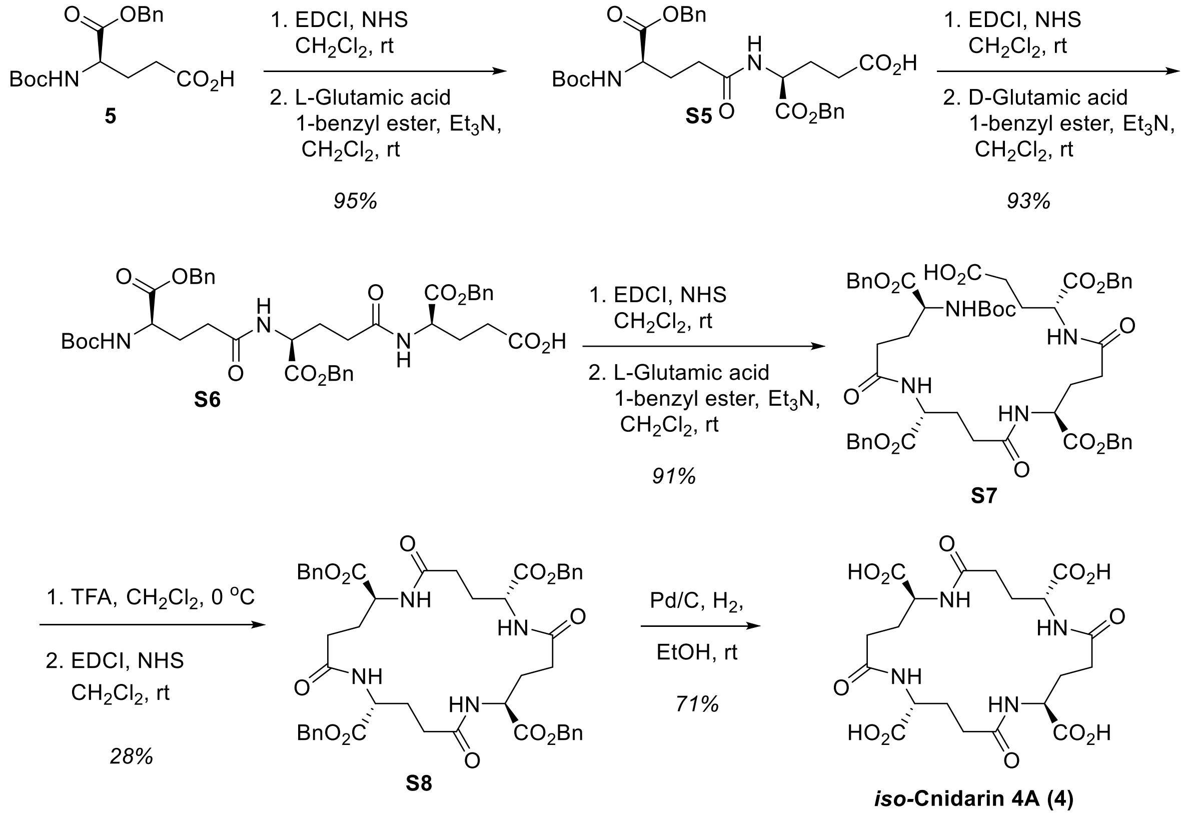

(S)-5-(Benzyloxy)-4-((R)-5-(benzyloxy)-4-((tert-butoxycarbonyl)amino)-5-oxopentanamido)-5-oxopentanoic acid (**S5**)

To a solution of **5** (1.808 g, 5.36 mmol) and *N*-hydroxysucinimide (0.864 g, 7.50 mmol) in dichloromethane (26.8 mL) at room temperature EDCI (1.28 g, 6.70 mmol) was added. The reaction was monitored by TLC and after 18 hr was diluted with dichloromethane (100 mL) and washed 3 × with saturated KH_2PO_4. The combined aqueous layers were back extracted with dichloromethane, and the combined organic layers were washed with saturated NaCl, dried over $MgSO_4$ and concentrated, providing the crude NHS ester (2.52 g, quant) as a white powder, deemed suitable for use in the subsequent reaction. To a solution of the crude NHS ester (1.566 g, 3.61 mmol) and triethylamine (0.704 mL, 0.511g, 5.05 mmol) in dichloromethane (36 mL) at room temperature, 1-benzyl L-glutamic acid (0.984 g, 4.15 mmol) was added. The reaction was monitored by TLC and after 22 hr was diluted with dichloromethane (114 mL) and washed 4 × with saturated KH_2PO_4. The combined aqueous layers were back extracted with dichloromethane and the combined organic layers were washed with saturated NaCl, dried over $MgSO_4$ and concentrated. The crude product was purified by flash chromatography on silica gel, (2% to 4% methanol in dichloromethane with 0.5% acetic acid acid) to provide **S5** (1.91 g, 95% (two step yield)) as a white powder: mp 89 - 91 °C; R*f* = 0.4 (5% methanol in dichloromethane with 0.5% acetic acid); ^1H NMR (300 MHz, CD_3OD) δ 7.36-

7.26 (m, 10H), 5.13 (s, 2H), 5.15 (s, 2H), 4.44 (dd, *J* = 6.0,9.0, 1H), 4.15 (dd, *J* = 3.0,9.0, 1H), 2.38-2.27 (m, 4H), 2.19-2.02 (m, 2H), 1.98-1.81 (m, 2H), 1.41 (s, 9H); ^{13}C NMR (300 MHz, CD_3OD) δ 174.8, 173.7, 172.4, 171.7,156.9, 136.0, 136.0, 128.4, 128.3, 128.1, 128.1, 128.0, 79.6, 66.9, 66.8, 53.7, 52.2, 31.9, 30.0, 27.7, 27.4, 26.5; IR 3362 (br), 2968 (m), 1722 (m), 1654 (s), 1519 (m), 1451 (s) cm^{-1}; TOF-MS *m/z* 579.2306 [M+Na]$^+$ ($C_{29}H_{36}N_2O_9Na^+$ requires 579.2319, Δ -1.3 mmu). See Figures S20 and S21 for ^{1}H and ^{13}C NMR spectra.

(6R,11S,16R)-6,11,16-Tris((benzyloxycarbonyl)-2,2-dimethyl-4,9,14-trioxo-3-oxa-5,10,15-triazanonadecan-19-oic acid (**S6**)

To a solution of **S5** (2.641 g, 4.75 mmol) and *N*-hydroxysucinimide (0.765 g, 6.64 mmol) in dichloromethane (24 mL) at room temperature, EDCI (1.14 g, 5.93 mmol) was added. The reaction was monitored by TLC and after 18 hr was diluted with dichloromethane (76 mL) and washed 4 × with saturated KH_2PO_4. The combined aqueous layers were back extracted with dichloromethane and the combined organic layers were washed with saturated NaCl, dried over $MgSO_4$ and concentrated, providing the crude NHS ester (3.19 g, quant) as a white foam deemed suitable for use in the subsequent reaction. To a solution of the crude NHS ester (1.48 g, 2.27 mmol) and triethylamine (0.443 mL, 0.321 g, 3.17 mmol) in dichloromethane (22.7 mL) at room temperature, 1-benzyl D-glutamate (0.619 g, 2.61 mmol) was added. The reaction was monitored by TLC and after 28 hr was diluted with dichloromethane (177 mL) and washed 4 × with saturated KH_2PO_4. The combined aqueous layers were back extracted with dichloromethane and the combined organic layers were washed with saturated NaCl, dried over $MgSO_4$ and concentrated. The crude product was purified by flash chromatography on silica gel, (5% methanol in dichloromethane with 0.5% acetic acid) to provide **S6** (1.62 g, 93% (two step yield)) as a white foam: R*f* = 0.33 (5% methanol in dichloromethane with 0.5% acetic acid); ^{1}H NMR (300 MHz, CD_3OD) δ 7.38-7.25 (m, 15H), 5.17-5.12 (m, 6H), 4.52-4.39 (m, 2H), 4.17 (dd, *J* = 6.0,9.0, 1H), 2.40-2.26 (m, 6H), 2.21-2.03 (m, 3H), 2.01-1.82 (m, 3H), 1.41 (s, 9H); ^{13}C NMR (300 MHz, CD_3OD) δ 174.8, 173.7, 173.6, 172.4, 171.7, 171.6, 156.8, 136.0, 136.0, 136.0, 128.35, 128.3, 128.3, 128.1, 128.0, 128.0, 128.0, 79.6, 66.9, 66.8, 53.7, 52.5, 52.3, 31.9, 31.8, 30.0, 27.7, 27.4, 27.2, 26.5; IR 3321 (br), 3046 (m), 2953 (m), 2356 (s), 1737 (s), 1654 (s), 1529 (s) cm^{-1}; TOF-MS *m/z* 798.3242 [M+Na]$^+$ ($C_{41}H_{49}N_3O_{12}Na^+$ requires 798.3214, Δ +2.8 mmu). See Figures S22 and S23 for ^{1}H and ^{13}C NMR spectra.

(6S,11R,16S,21R)-6,11,16,21-Tetrakis((benzyloxy)carbonyl)-2,2-dimethyl-4,9,14,19-tetraoxo-3-oxa-5,10,15,20-tetraazatetracosan-24-oic acid (**S7**)

To a solution of **S6** (1.489 g, 1.92 mmol) and *N*-hydroxysucinimide (0.309 g, 2.69 mmol) in dichloromethane (19.2 mL), EDCI (0.460 g, 2.40 mmol) was added. The reaction was monitored by TLC and after 27 hr was diluted with dichloromethane (181 mL) and washed 4 × with saturated KH_2PO_4. The combined aqueous layers were back extracted with dichloromethane and the combined organic layers were washed with saturated NaCl, dried over $MgSO_4$ and concentrated, providing the crude NHS ester (1.73 g, quant) as a white foam deemed suitable for use in the subsequent reaction. To a solution of **the crude** (1.537 g, 1.76 mmol) and triethylamine (0.343 mL, 0.249 g, 2.46 mmol) in dichloromethane (17.6 mL) at room temperature, 1-benzyl D-glutamate (0.480 g, 2.02 mmol) was added. The reaction was monitored by TLC and after 22 hr was diluted with dichloromethane (183 mL) and washed 4 × with saturated KH_2PO_4. The combined aqueous layers were back extracted with

dichloromethane and the combined organic layers were washed with saturated NaCl, dried over $MgSO_4$ and concentrated. The crude product was further purified by flash chromatography on silica gel (eluting with 4% methanol in dichloromethane and 0.5% acetic acid) to provide **S7** (1.60 g, 91% (two step yield)) as a white foam: R*f* = 0.35 (5% methanol in dichloromethane with 0.5% acetic acid); ^{1}H NMR (300 MHz, CD_3OD) δ 7.40-7.26 (m, 20H), 5.18-5.13 (m, 8H), 4.51-4.39 (m, 3H), 4.18 (dd, *J* = 3.0,6.0, 1H), 2.41-2.26 (m, 8H), 2.23-2.06 (m, 4H), 2.03-1.84 (m, 4H), 1.43 (s, 9H); ^{13}C NMR (300 MHz, CD_3OD) δ 174.8, 173.7, 173.6, 172.4, 171.8, 171.7, 171.6, 156.8, 136.0, 135.9, 128.4, 128.4, 128.1, 128.0, 128.0, 128.0, 79.7, 66.9, 66.8, 53.8, 52.5, 52.4, 52.3, 31.9, 31.8, 31.8, 30.0, 27.7, 27.4, 27.3, 27.1, 26.5; IR 3321 (br), 3061 (m), 2968 (m), 1737 (s), 1654 (s), 1529 (s) cm^{-1}; TOF-MS *m*/*z* 1017.4101 [M+Na]$^+$ ($C_{53}H_{62}N_4O_{15}Na^+$ requires 1017.4109, Δ -0.8 mmu). See Figures S24 and S25 for ^{1}H and ^{13}C NMR spectra.

Tetrabenzyl (2S,7R,12S,17R)-5,10,15,20-tetraoxo-1,6,11,16-tetraazacycloicosane-2,7,12,17-tetracarboxylate (**S8**)

To a solution of **S7** (0.495 g, 0.498 mmol) in dichloromethane (25 mL) at 0 ºC, a pre-cooled 1:1 solution of trifluoroacetic acid and dichloromethane (25 mL) was added portionwise; 5.0 mL were added every 5 min (5 X) for a total of 25 mL over 25 min. The reaction was monitored TLC by conducting "mini workups" (drying under nitrogen gas followed by reconstituting with dichloromethane, 3 × in total). After 1.5 hr the solvent was removed by rotary-evaporation, and the crude was reconstituted in dichloromethane followed again by evaporation (3 × in total). The crude was purified by flash chromatography on silica gel (4% to 10% methanol in dichloromethane) to provide the free ammonium trifluoroacetate (0.355 g, 71%) as a brown foam: R*f* = 0.24 (8% methanol in dichloromethane); ^{1}H NMR (300 MHz, CD_3OD) δ7.41-7.22 (m, 20H), 5.245 (d, *J*=3.0, 2H), 5.15-5.10 (m, 6H), 4.49-4.36 (m, 3H), 4.11 (t, *J*=12.0, 1H), 2.45 (t, *J*=15.0, 2H), 2.37-2.24 (m, 6H), 2.23-2.02 (m, 5H), 2.00-1.81 (m, 3H); ^{13}C NMR (300 MHz, CD_3OD) δ 174.8, 173.6, 173.5, 172.9, 171.7, 171.6, 186.8, 135.9, 135.9, 135.1, 128.6, 128.5, 128.4, 128.4, 128.3, 128.2, 128.1, 128.01, 128.0, 68.2, 67.0, 66.9, 66.9, 52.5, 52.3 31.7, 30.8, 29.9, 27.1, 26.5, 25.9; IR 3310 (br), 3046 (m), 2947 (m), 1737 (s), 1659 (s), 1540 (s) cm^{-1}. To a solution of the free ammonium trifluoroacetate (0.151 g, 0.149 mmol) and triethylamine (6.36 μL, 4.61 mg, 0.456 mmol) in dichloromethane (152 mL), EDCI (0.0874 g, 0.456 mmol) was added. The reaction was monitored by TLC and after 17 hr was diluted with dichloromethane (75 mL) and washed 4 × with saturated KH_2PO_4. The combined aqueous layers were back extracted with dichloromethane, and the combined organic layers were washed with saturated NaCl, dried over $MgSO_4$ and concentrated. The crude product was purified by recrystallization according to the following procedure. The crude sample of was dissolved in warm dichloromethane, filtered through cotton in a glass funnel followed by the addition of a small volume of hexane. The solution was placed in a fume hood overnight and then in a freezer for 4 days providing fine crystals. The suspension was centrifuged, and the supernatant removed followed by 2 hexane-wash / centrifugation cycles providing **S8** (0.052 g, 40%) as a white powder: mp 248 - 250 °C; ^{1}H NMR (300 MHz, $CDCl_3$) δ7.38-7.27 (m, 20H), 7.17 (d, *J*=6.0, 4H), 5.19-5.08 (m, 8H), 4.70-4.60 (m, 4H), 2.48-2.35 (m, 4H), 2.26-2.05 (m, 12H); ^{13}C NMR (300 MHz, $CDCl_3$) δ 173.1, 173.1, 171.0, 135.4, 128.8, 128.6, 128.3, 67.5, 53.4, 53.3, 33.3, 27.6, 27.5; IR 3290 (br), 3072 (m), 2937 (m), 1732 (s), 1649 (s), 1550 (s) cm^{-1}; TOF-MS *m*/*z* 899.3461 [M+Na]$^+$ ($C_{48}H_{52}N_4O_{12}Na^+$ requires 899.3479, Δ -1.8 mmu). See Figures S26 and S27 for ^{1}H and ^{13}C NMR spectra.

2.1,5.4-Anhydro(γ-L-glutamyl-γ-D-glutamyl-γ-L-glutamyl-D-glutamic acid) (iso-cnidarin 4A, **4**)

Above a solution of **S8** (0.0101 g, 0.0115 mmol) and 10% Pd/C (~2 mg) in ethanol (5.0 mL) at room temperature, hydrogen gas was maintained at ambient pressure for 21 hr. The reaction was then filtered through celite followed by methanol washing (3 × 1 mL), methanol/water 1:1 (3 × 1 mL) then water (3 × 1 mL). The crude product was purified by HPLC using a Waters Atlantis dC18 (10 × 250 mm, 10 μm particle size) column (mobile phase A: water/formic acid (1000:1); mobile phase B: acetonitrile/formic acid (1000:1); gradient: 2.5% B, 0-7 min, 2.5–100% B, 7-27 min, 100% B, 27-35 min) to provide **4** (0.042 g, 71%) as a white solid: decomp = 277 - 283 °C; ^{1}H NMR (500 MHz, D_2O) δ4.295 (dd, *J*=5.0,10, 4H), 2.49-2.40 (m, 4H), 2.37-2.28 (m, 4H), 2.19-2.10 (m, 4H), 2.06-1.95 (m, 4H); ^{13}C NMR (500 MHz, D_2O) δ 178.3, 177.9, 55.8, 34.9, 29.0; IR 3312 (br), 2358 (m), 1644 (m), 1594 (s), 1410 (br) cm^{-1}; TOF-MS *m*/*z* 539.1602 [M+Na]$^+$ ($C_{20}H_{28}N_4O_{12}Na^+$ requires 539.1601, Δ +0.1 mmu). See Figures S28 and S29 for ^{1}H and ^{13}C NMR spectra.

Section S3. Chromatography and chemical analyses of cnidarin 4A (1)

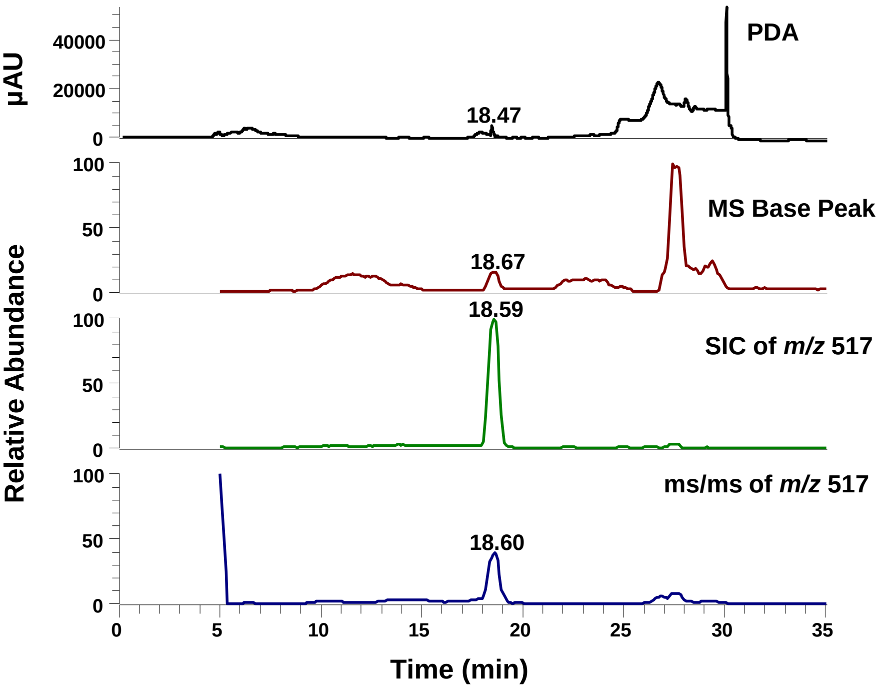

Figure S1. LC-DAD-MS of *Alatina alata* venom. Chromatograms shown from top to bottom panel are: photo diode array detector (PDA) UV absorption (200-600 nm), MS base peak (*m*/*z* 100-1000), extracted ion chromatograms for *m*/*z* 517 ion and MS/MS product ions for *m*/*z* 517. Extract was analyzed on a Waters Atlantis dC18 (3.0 x 250 mm column, 5 µm particle size) column (mobile phase A: water/formic acid (1000:1); mobile phase B: acetonitrile/formic acid (1000:1); gradient: 0% B, 0-10 minutes, 0–50% B, 10-20 minutes, 50-100% B, 20-23 minutes, 100% B, 23-25 minutes). The peak at 18.6 min in extracted ion ($[M+H]^+$ *m*/*z* 517) and MS/MS chromatograms (bottom 2 panels) was a mixture resolved with LC conditions described in Figure S2.

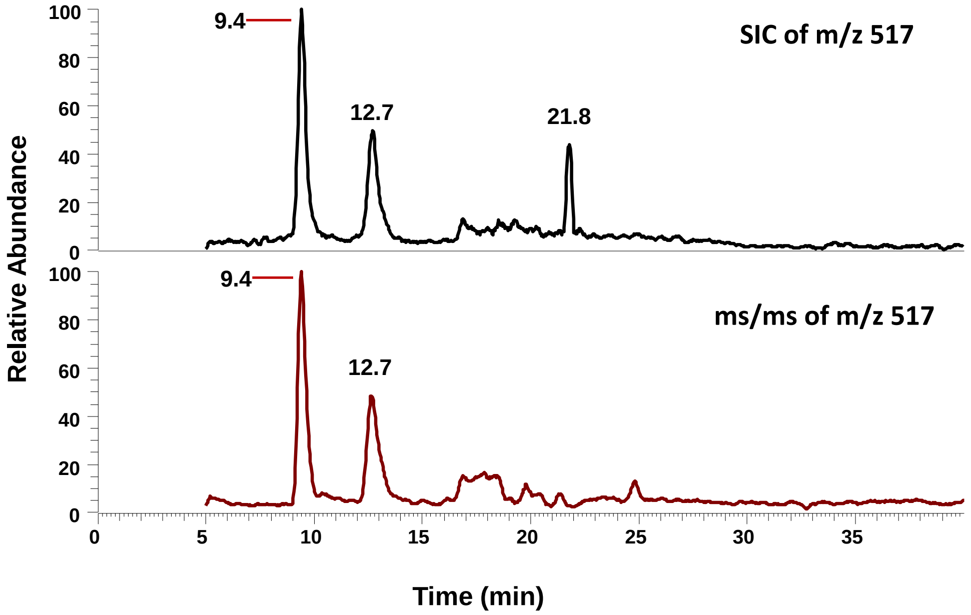

Figure S2. Extracted [M+H]$^+$ ion (*m/z* 517) and MS/MS chromatograms of crude cnidarin 4A (**1**) (see peak at 18.6 min in Figure S1) from *Alatina alata* venom. Sample was reconstituted in 1% formic acid analyzed on a Waters Atlantis dC18 column (3.0 x 250 mm, 5 µm particle size) (mobile phase A: water/formic acid (1000:1); mobile phase B: acetonitrile/formic acid (1000:1); gradient: 2.5% B, 0-7 minutes, 2.5–100% B, 7-27 minutes, 100% B, 27-35 minutes). Compound **1** eluted at 9.4 min.

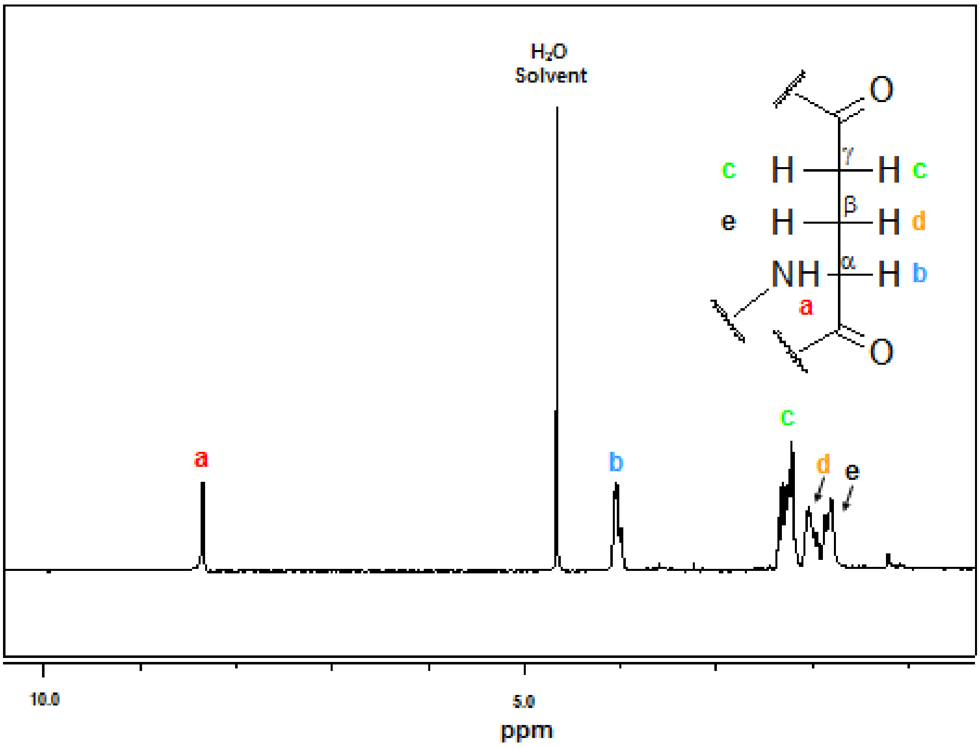

Figure S3. Capillary ^{1}H nuclear magnetic resonance (NMR) spectrum (500 MHz, D_2O/H_2O) of crude cnidarin 4A (**1**). Spectral data indicate multiple glutamic acids.

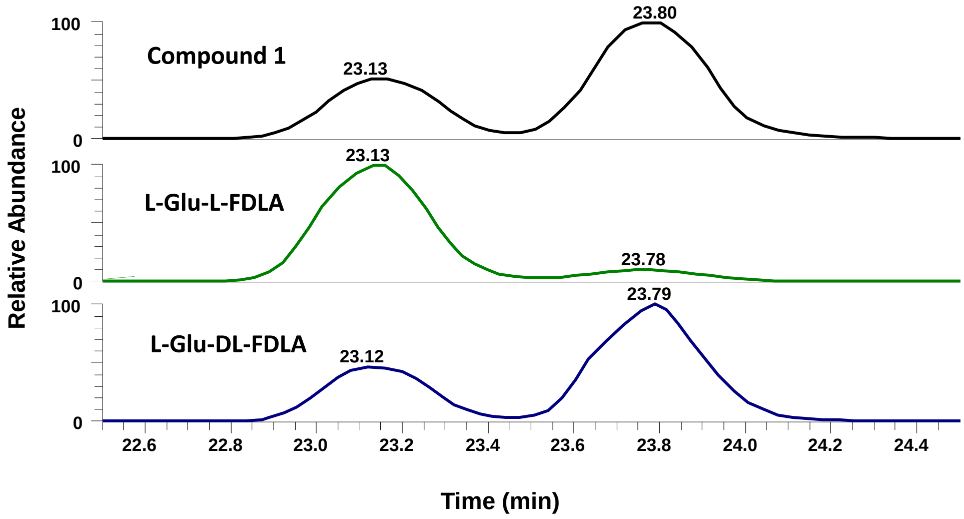

Figure S4. LC-MS of FDLA-labeled products of the compound **1** hydrolysate, L-glutamic acid, and DL-glutamic acid. Chromatograms are summed extracted ion chromatograms of *m*/*z* of 442 and 883 ions, which correspond to protonated glutamic acid-FDLA monomer and dimer ions. Products were analyzed using a Phenomenex Luna C18(2) (2.0 x 250 mm, 3 μm particle size) column (mobile phase A: water/formic acid (1000:1); mobile phase B: acetonitrile/formic acid (1000:1); gradient: 20-80% B, 0-30 minutes, 80% B, 30-35 minutes).

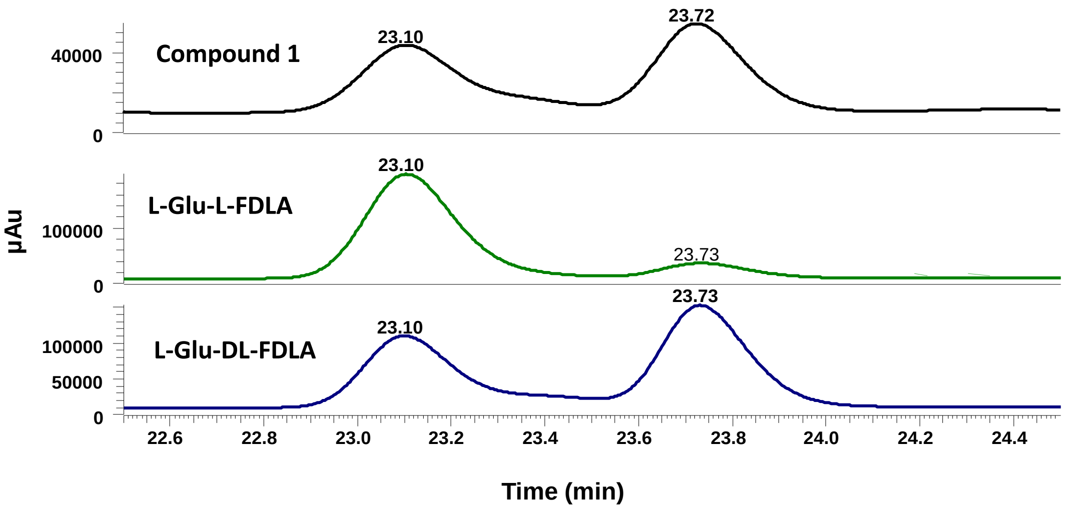

Figure S5. HPLC with PDA detection (200-600 nm) of FDLA-labeled products of compound **1** hydrolysate. (UV chromatograms from Figure S4). Products were analyzed on a Phenomenex Luna C18(2) (2.0 × 250 mm, 3 µm particle size) column (mobile phase A: water/formic acid (1000:1); mobile phase B: acetonitrile/formic acid (1000:1); gradient: 20-80% B, 0-30 min, 80% B, 30-35 min).

Table S1. Ratio of glutamic acid-FDLA derivatives of hydrolysate of compound **1** using HPLC with MS and UV detection.

Detector	relative concentration	
	D-glutamic acid	L-glutamic acid
UV	0.86	1.0
MS	0.99	1.0

Ratios were calculated from extracted ion chromatograms, see Figures S4 and S5.

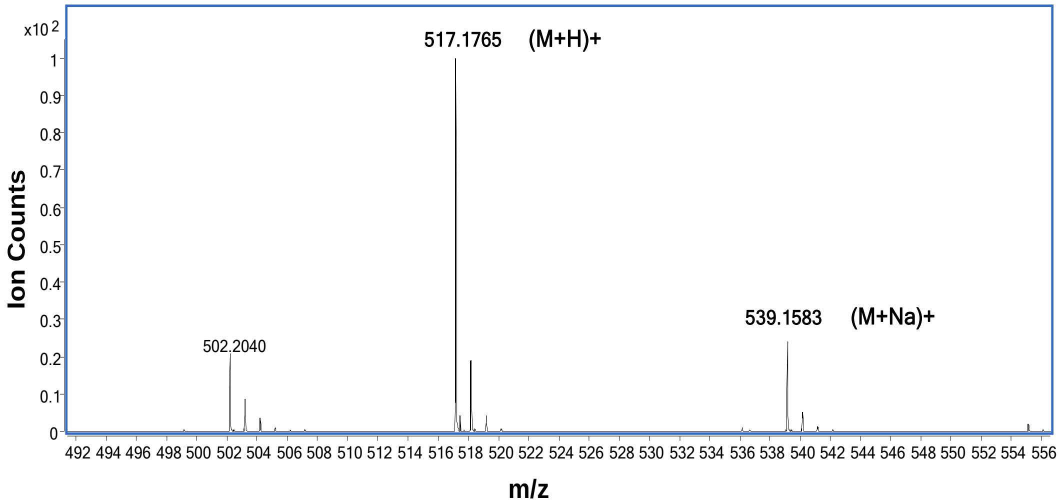

Figure S6. High resolution time-of-flight mass spectrometry (TOF-MS-ESI positive mode) of compound **1** from *Alatina alata* venom.

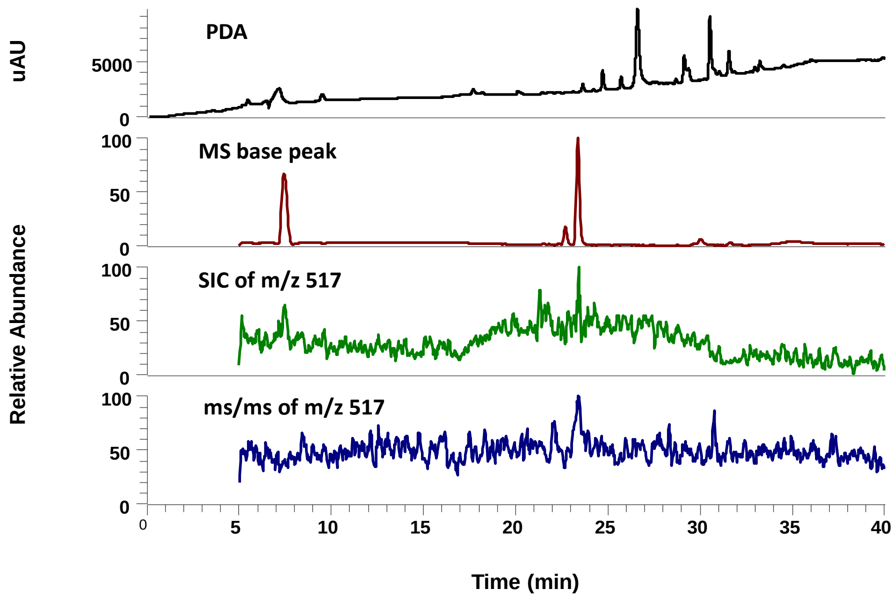

Figure S7. LC-DAD-MS of *Alatina alata* tentacle extract without nematocysts (panels from top to bottom: photo diode array UV detector, MS base peak, extracted ion chromatograms for the $[M+H]^+$ ion, *m*/*z* 517, and MS/MS product ions for *m*/*z* 517).

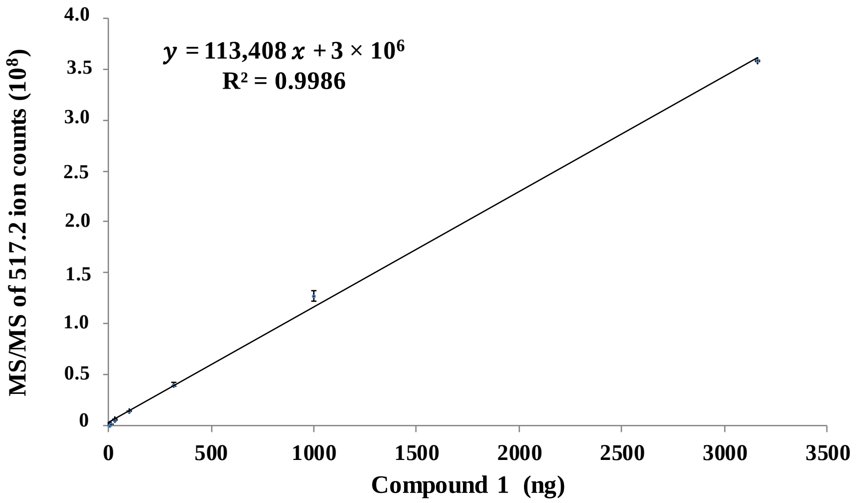

Figure S8. Calibration curve for MS/MS detection of cnidarin 4A (**1**) (product ion of *m*/z 517 fragmentation). Synthesized **1** was analyzed at semi log concentrations in triplicate with a fixed volume injection (0.343 – 343 ng on column). LC-MS conditions: Waters Atlantis dC18 column (3.0 × 250 mm, 5 μm particle size); mobile phase A: water/formic acid (1000:1); mobile phase B: acetonitrile/formic acid (1000:1); gradient: 2.5% B, 0-7 min, 2.5–100% B, 7-27 min, 100% B, 27-35 min).

Section S4. Cell toxicity and hemolytic activity of cnidarin 4A (1).

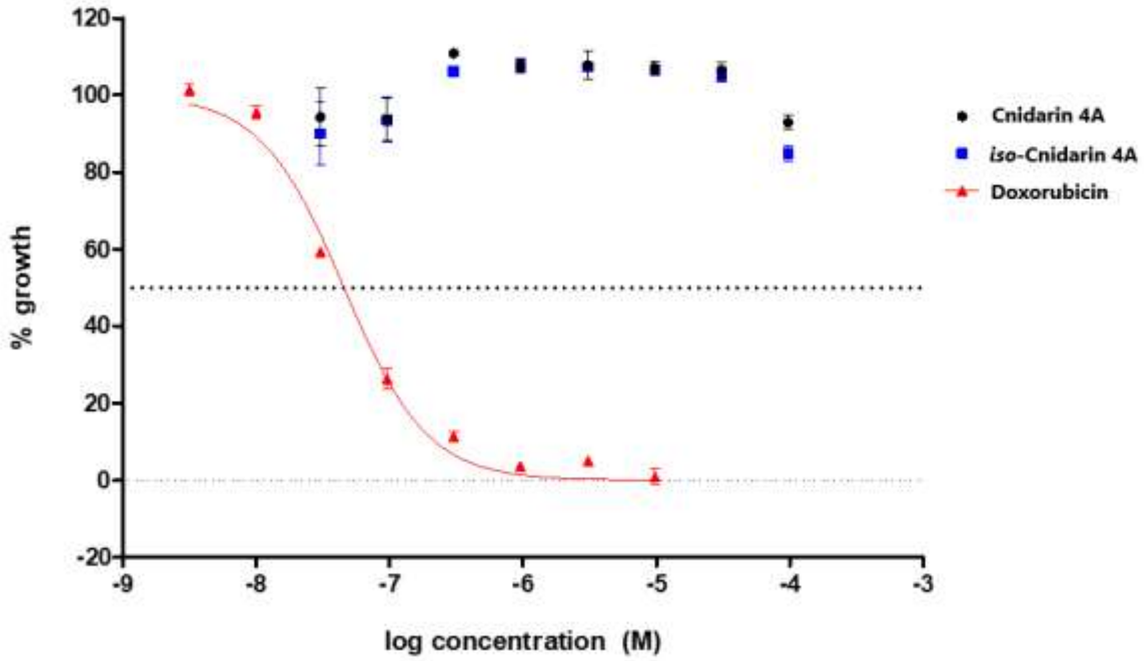

Figure S9. Cytotoxicity of doxorubicin, synthetic cnidarin 4A (**1**) and *iso*-cnidarin 4A (**4**) against HEK-293 cells. Doxorubicin is a positive control. Methods described in article.

Table S2. Hemolysis of red blood cells by cnidarin 4A (**1**) normalized to 1% TritonX-100 hemolysis.

treatment	% hemoglobin	Standard deviation
vehicle	2.63	0.31
24 µM cmpd **1**	3.74	1.04
121 µM cmpd **1**	2.86	0.20
TritonX-100, 1%	100	0.28

Treatments were tested in triplicate.

Section S5. NMR spectra of synthetic cnidarin 4A (1) and intermediates 8-12.

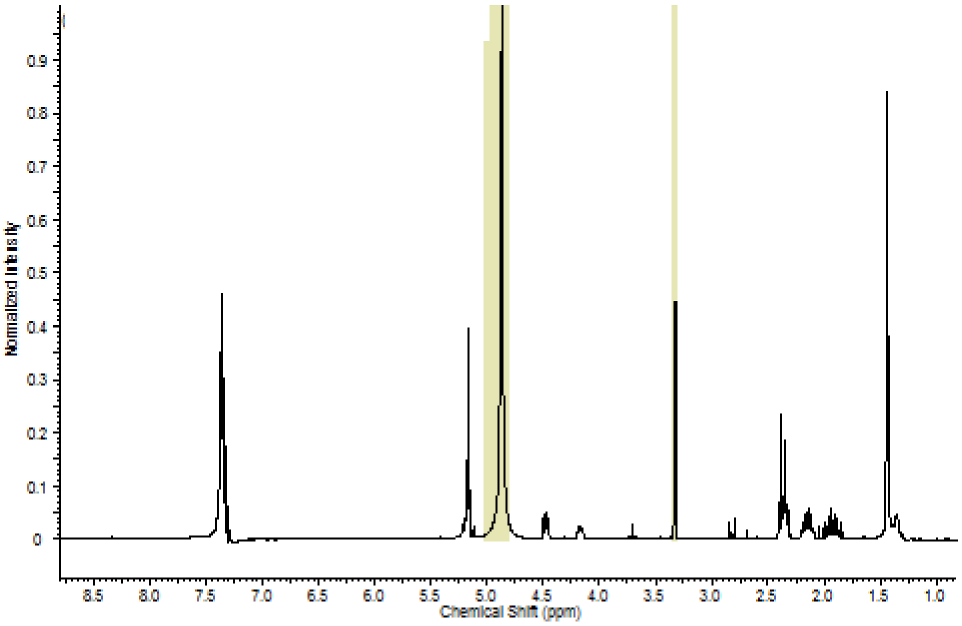

Figure S10. ^{1}H NMR (CD_3OD) spectrum for **6**.

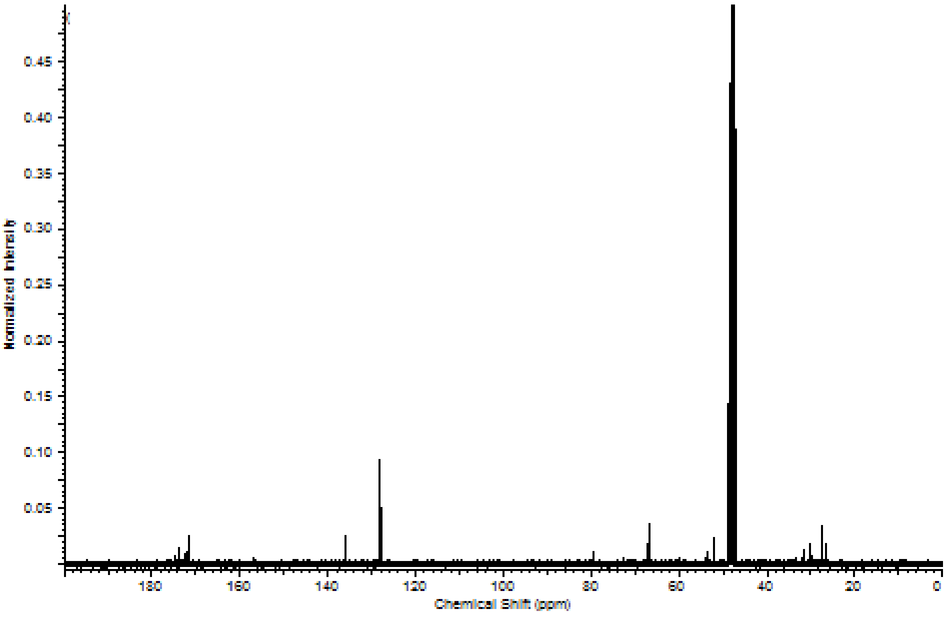

Figure S11. ^{13}C NMR (CD_3OD) spectrum for **6**.

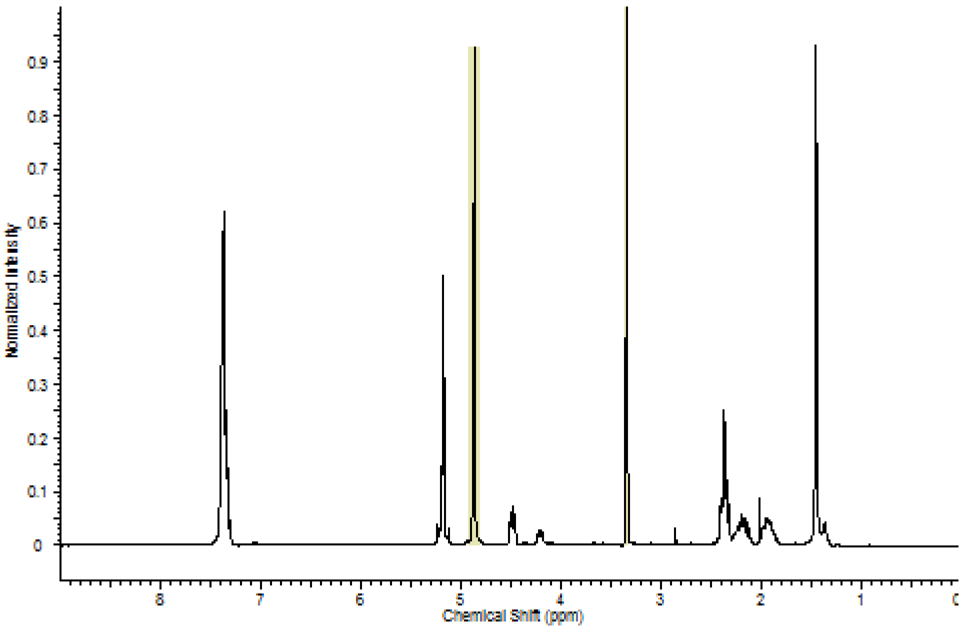

Figure S12. ^{1}H NMR (CD_3OD) spectrum for **7**.

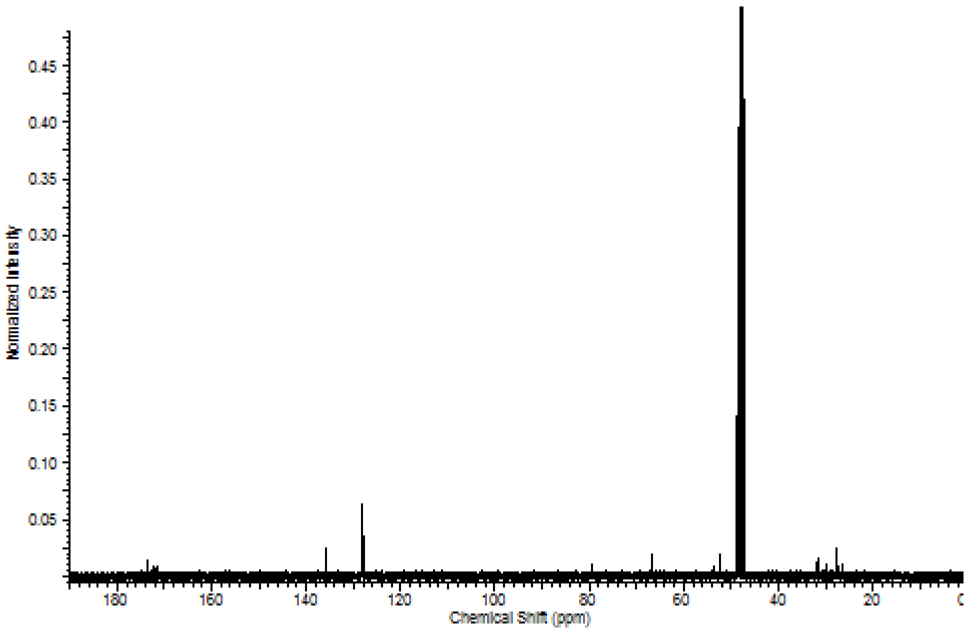

Figure S13. ^{13}C NMR (CD_3OD) spectrum for **7**.

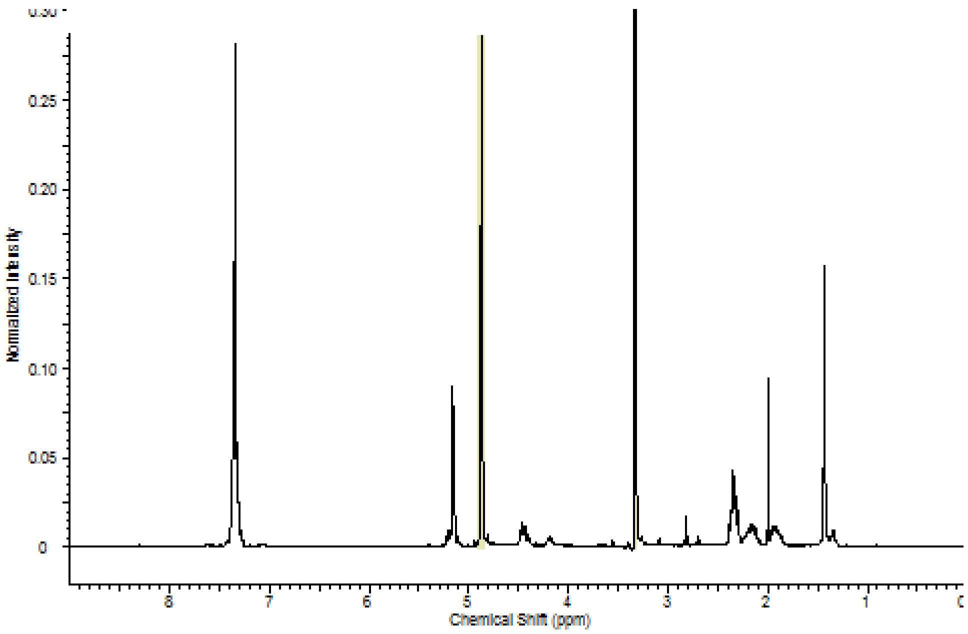

Figure S14. ^{1}H NMR (CD_3OD) spectrum for **8**.

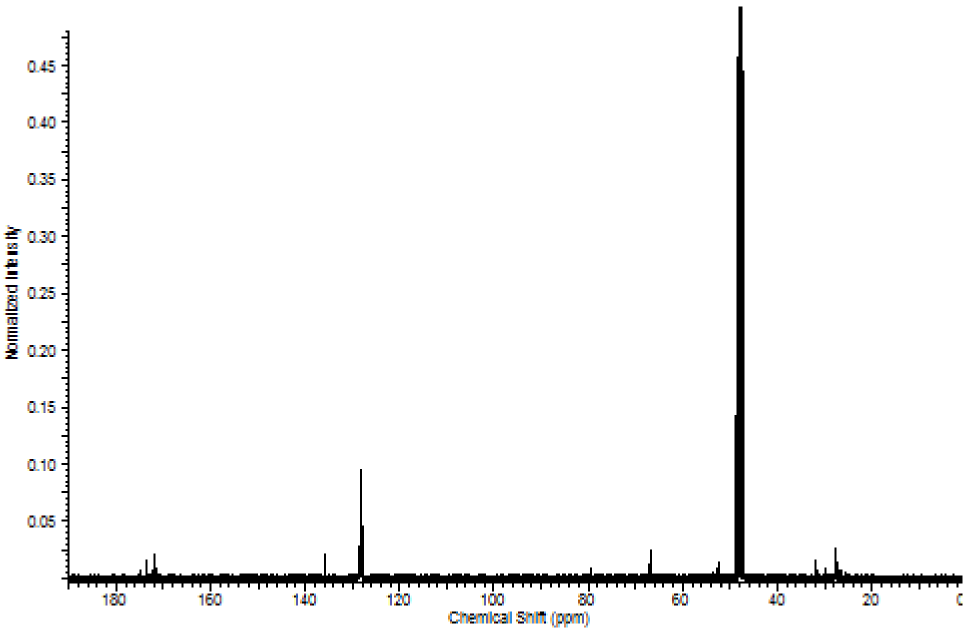

Figure S15. ^{13}C NMR (CD_3OD) spectrum for **8**.

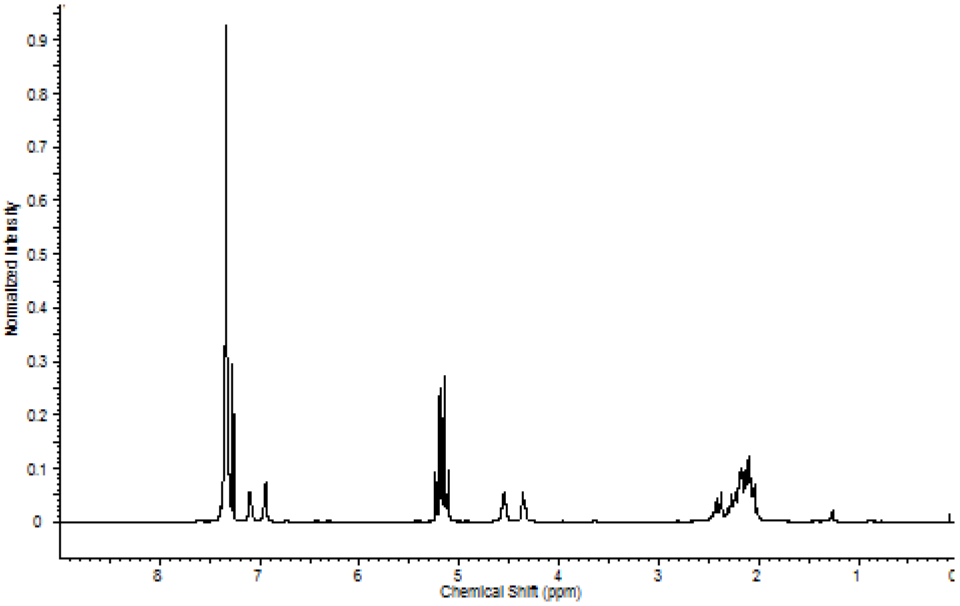

Figure S16. ^{1}H NMR ($CDCl_3$) spectrum for **9**.

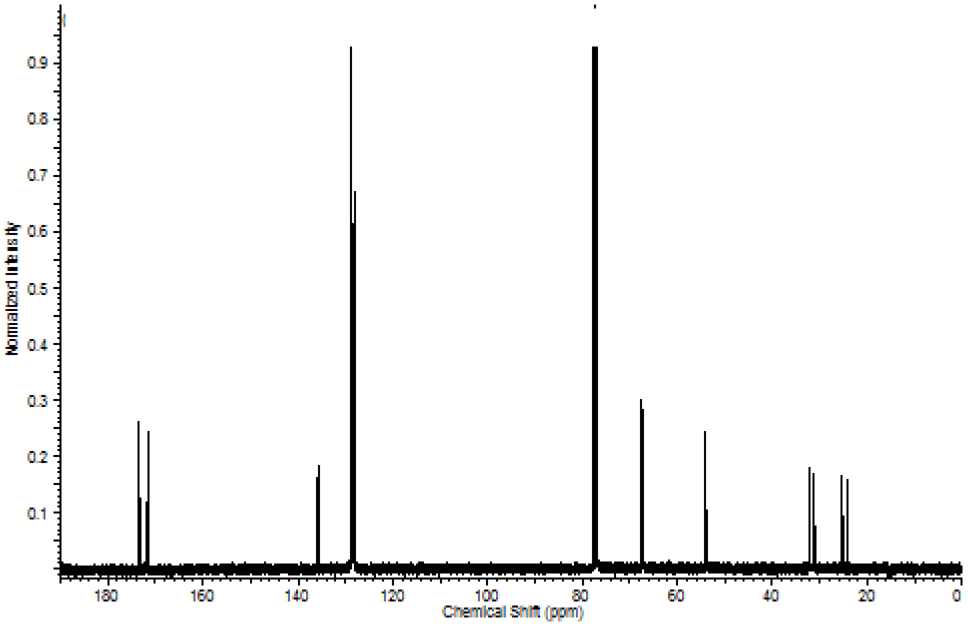

Figure S17. ^{13}C NMR ($CDCl_3$) spectrum for **9**.

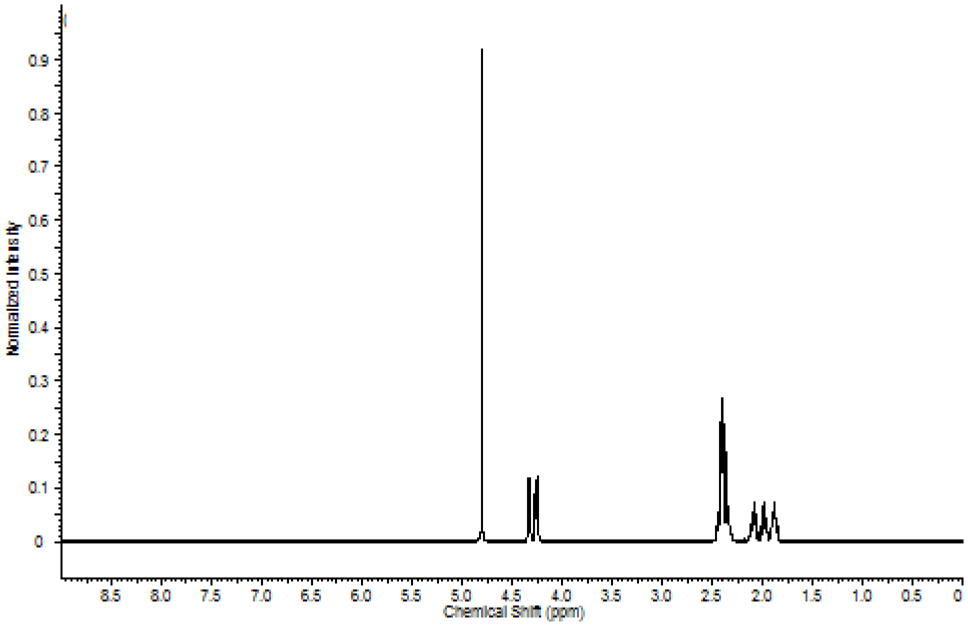

Figure S18. ^{1}H NMR (D_2O) spectrum for **1**.

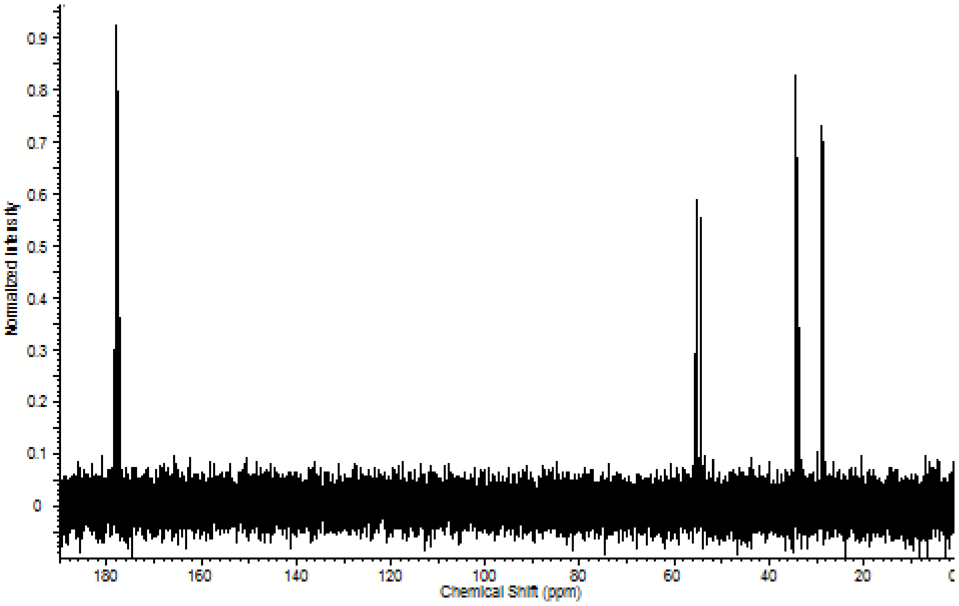

Figure S19. ^{13}C NMR (D_2O) spectrum for **1**.

Section S6. NMR spectra of synthetic *iso*-cnidarin 4A (4) and intermediates S5-S8.

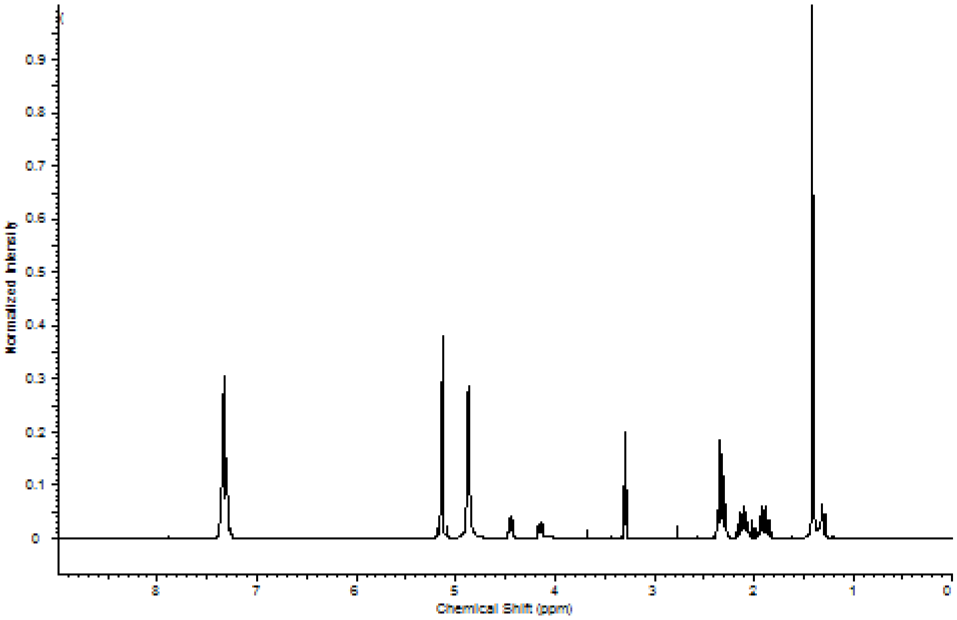

Figure S20. ^{1}H NMR (CD_3OD) spectrum for **S5**.

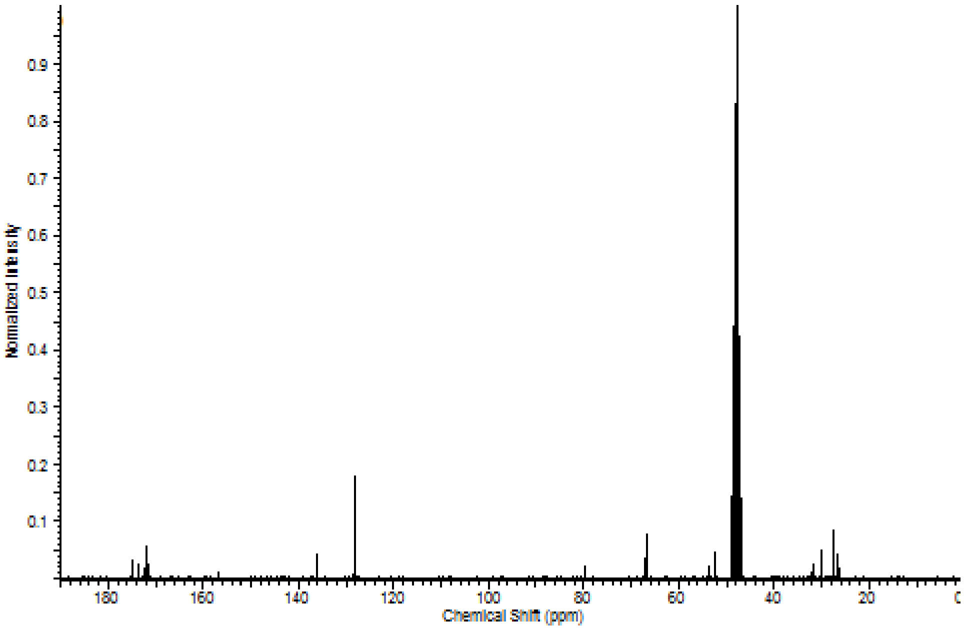

Figure S21. ^{13}C NMR (CD_3OD) spectrum for **S5**.

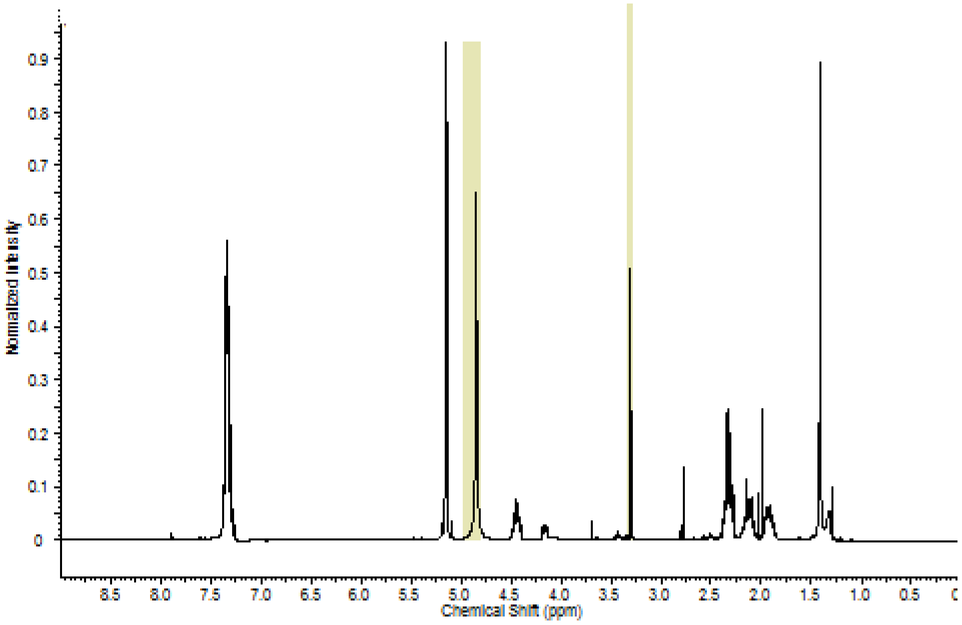

Figure S22. ^{1}H NMR (CD_3OD) spectrum for **S6**.

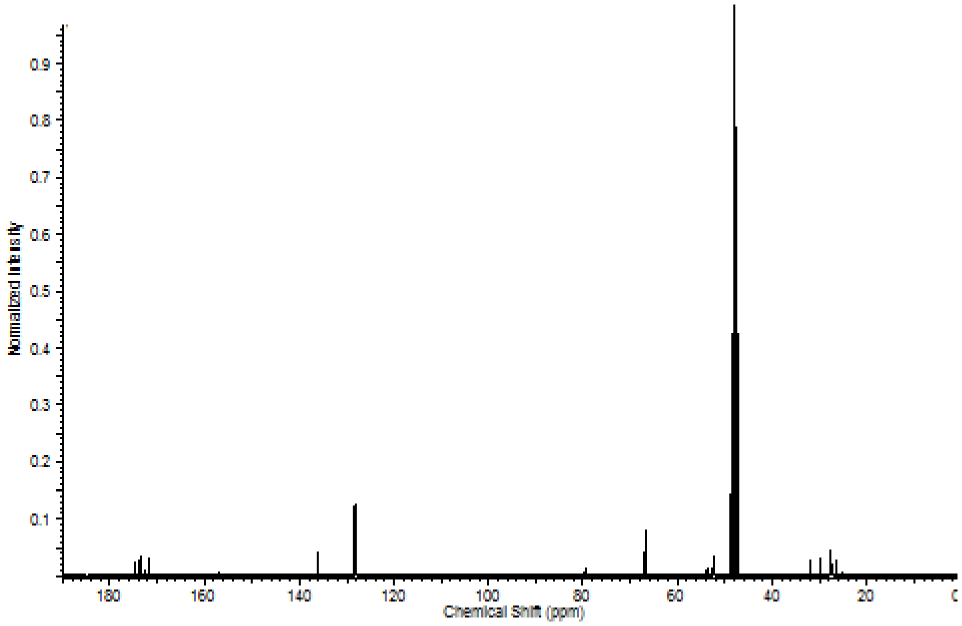

Figure S23. ^{13}C NMR (CD_3OD) spectrum for **S6**.

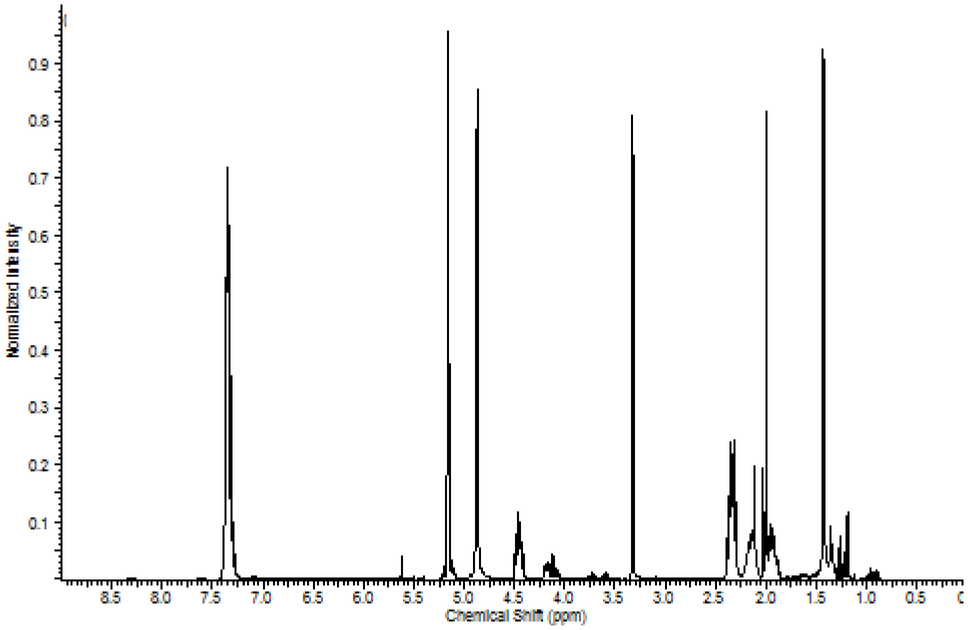

Figure S24. ^{1}H NMR (CD_3OD) spectrum for **S7**.

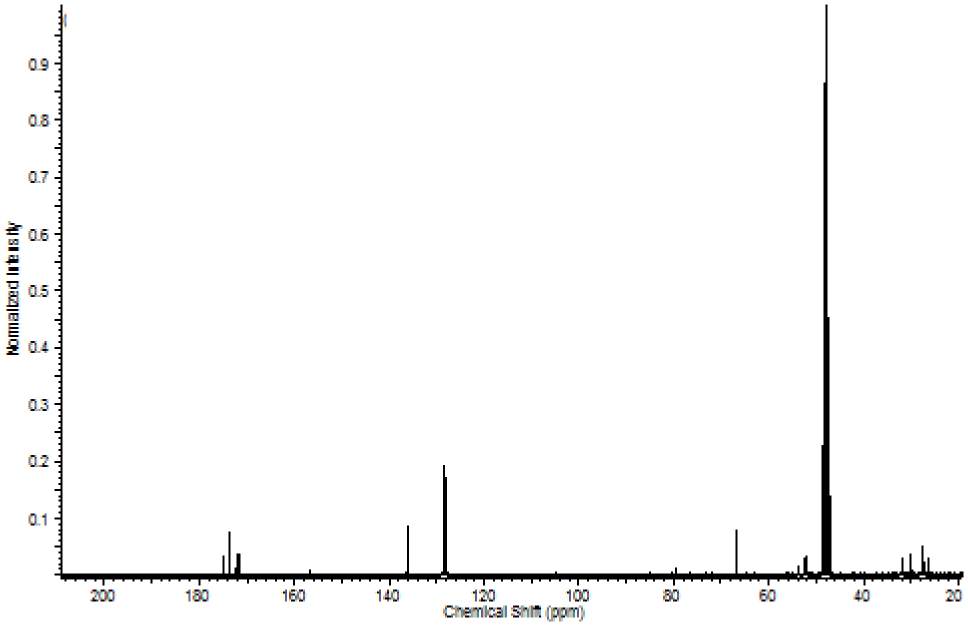

Figure S25. ^{13}C NMR (CD_3OD) spectrum for **S7**.

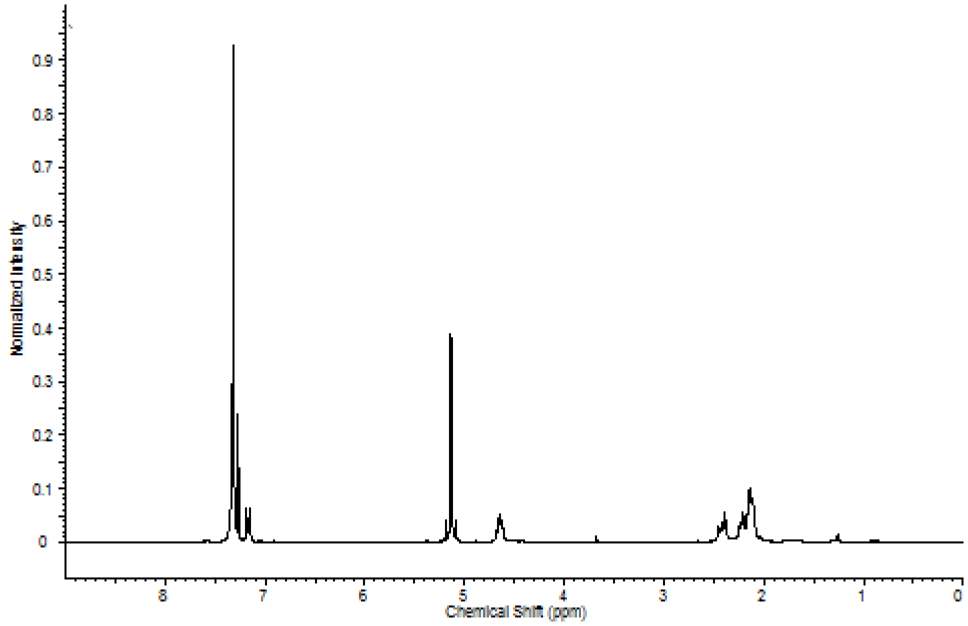

Figure S26. ^{1}H NMR ($CDCl_3$) spectrum for **S8**.

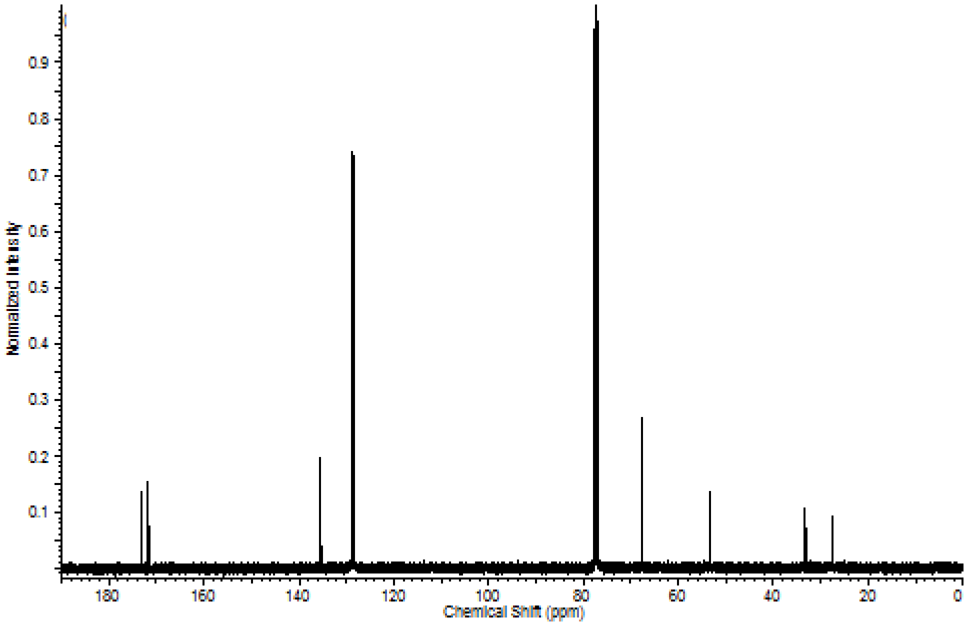

Figure S27. ^{13}C NMR ($CDCl_3$) spectrum for **S8**.

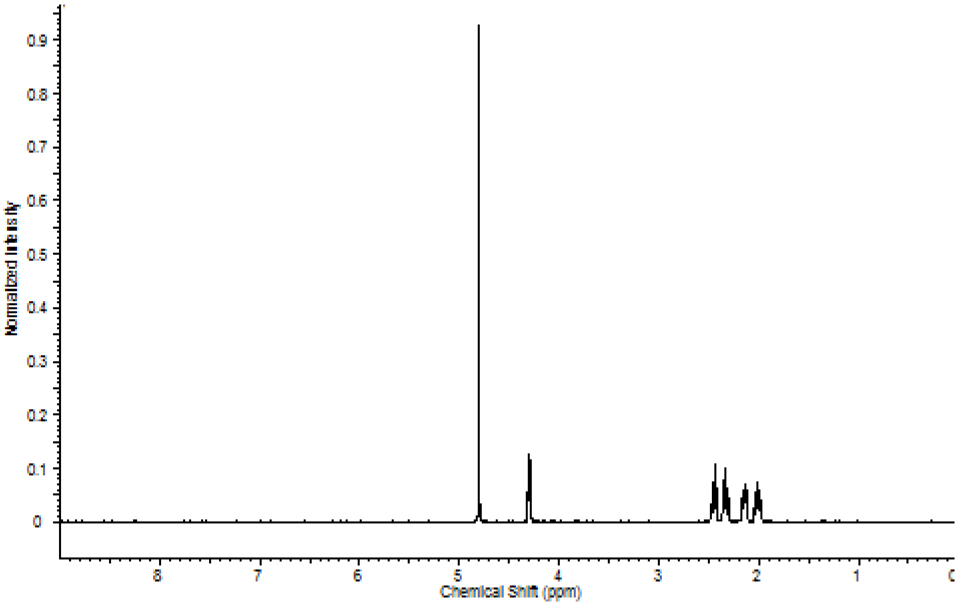

Figure S28. ^{1}H NMR (D_2O) spectrum for **4**.

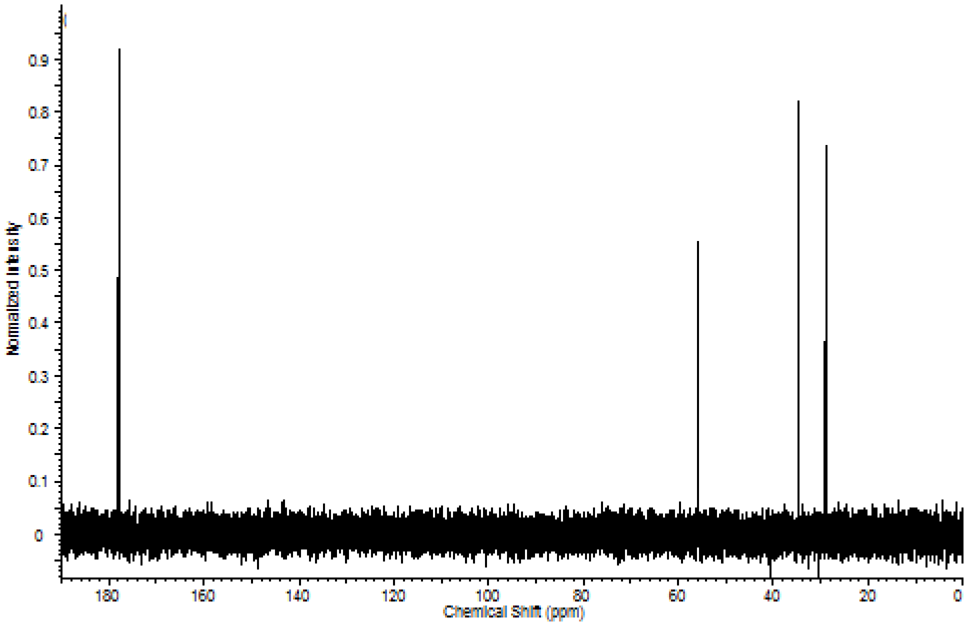

Figure S29. ^{13}C NMR (D_2O) spectrum for **4**.

Section S7. NMR spectra of synthetic cnidarin 4C (3) and intermediates.

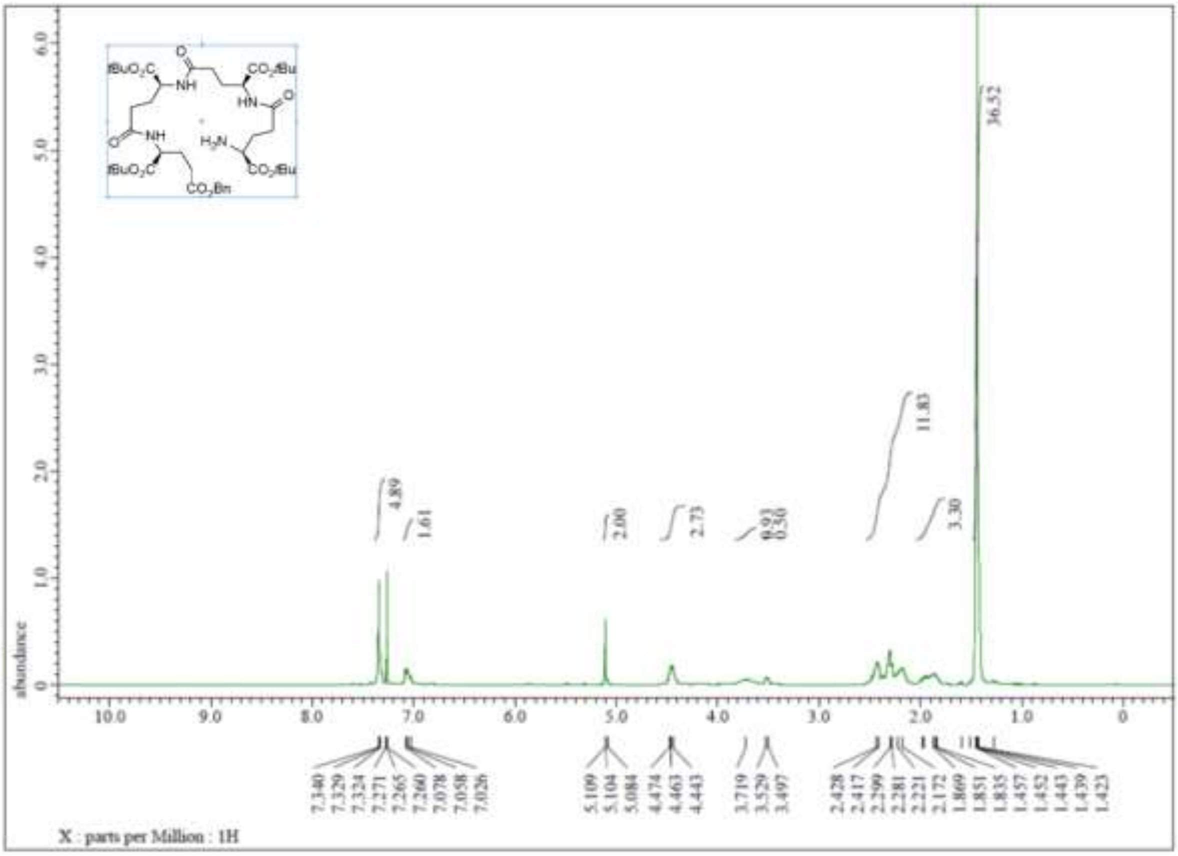

Figure S30. ^{1}H NMR ($CDCl_3$) spectrum for linear LLLL-glutamic acid with free amine.

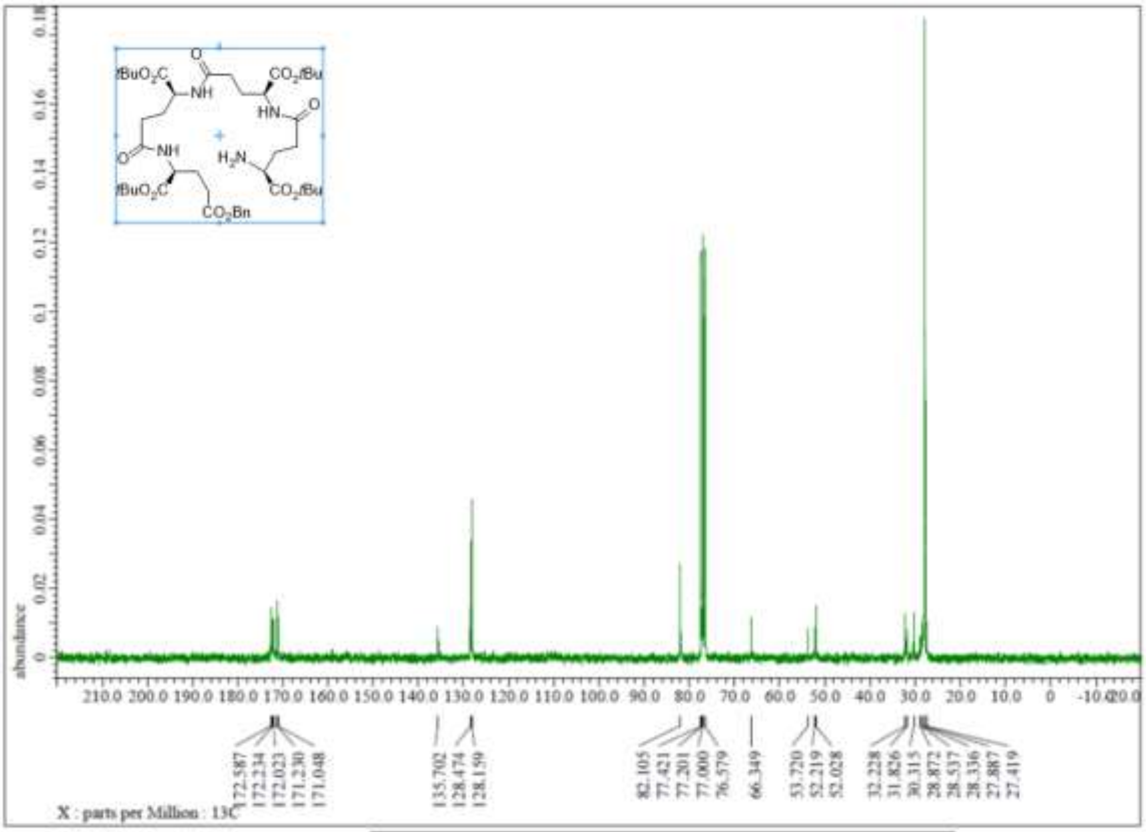

Figure S31. ^{13}C NMR ($CDCl_3$) spectrum for linear LLLL-glutamic acid with free amine.

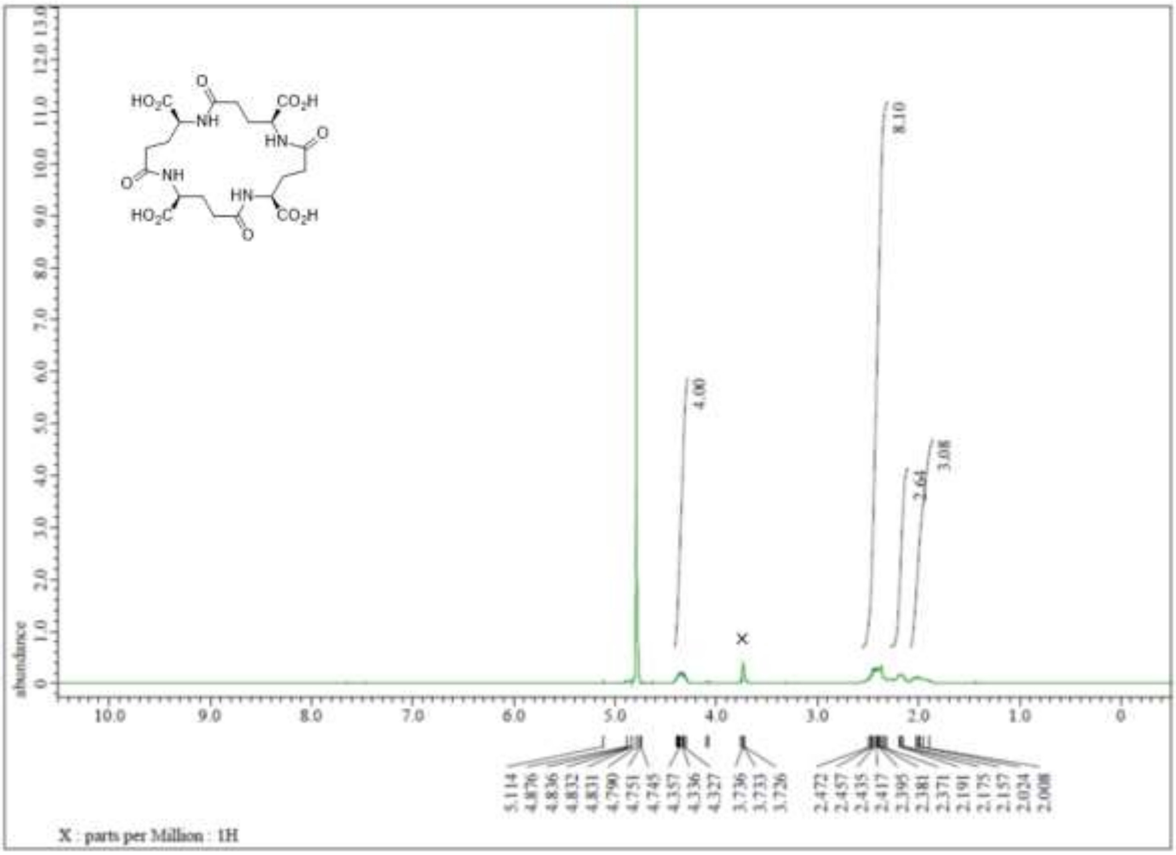

Figure S32. ^{1}H NMR (D_2O) spectrum for **3**.

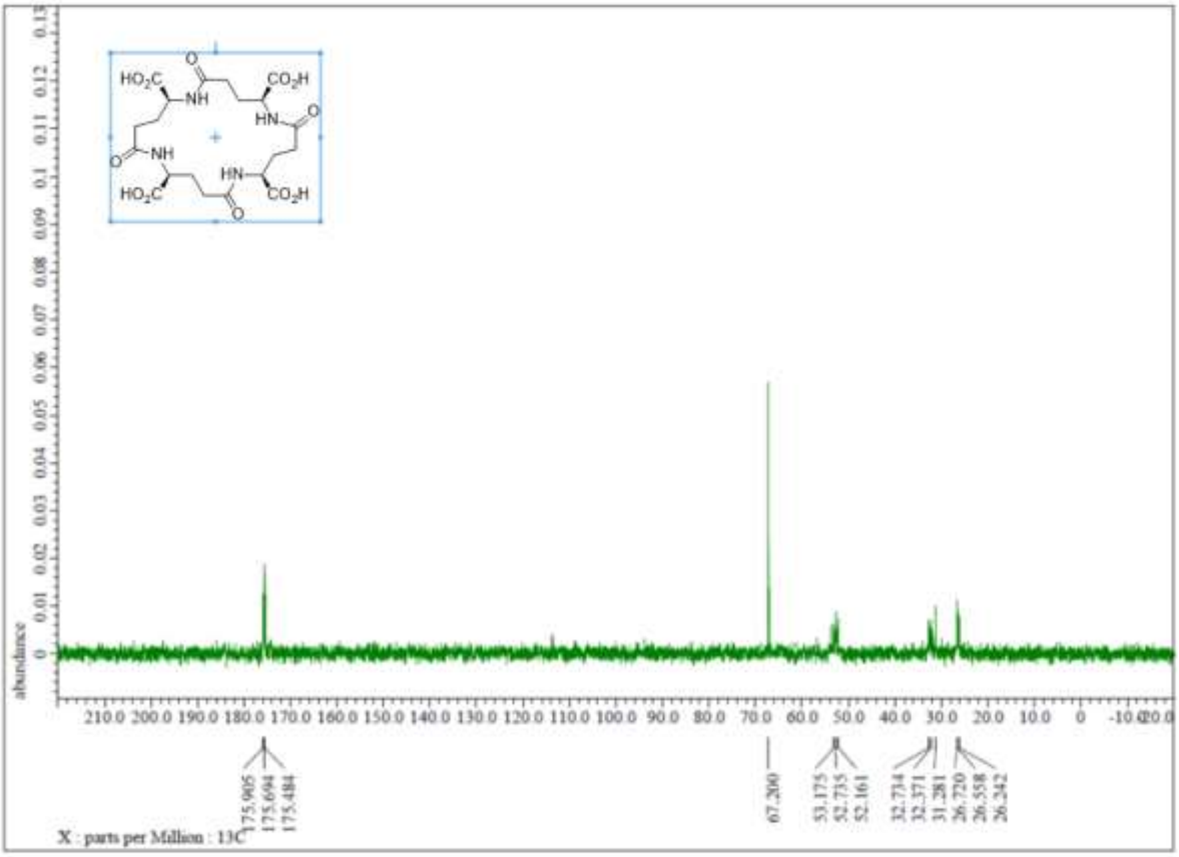

Figure S33. ^{13}C NMR (D_2O) spectrum for **3**; 1,4-dioxane added as internal standard.

Section S8. NMR spectra of synthetic cnidarin 4B (2) and intermediates.

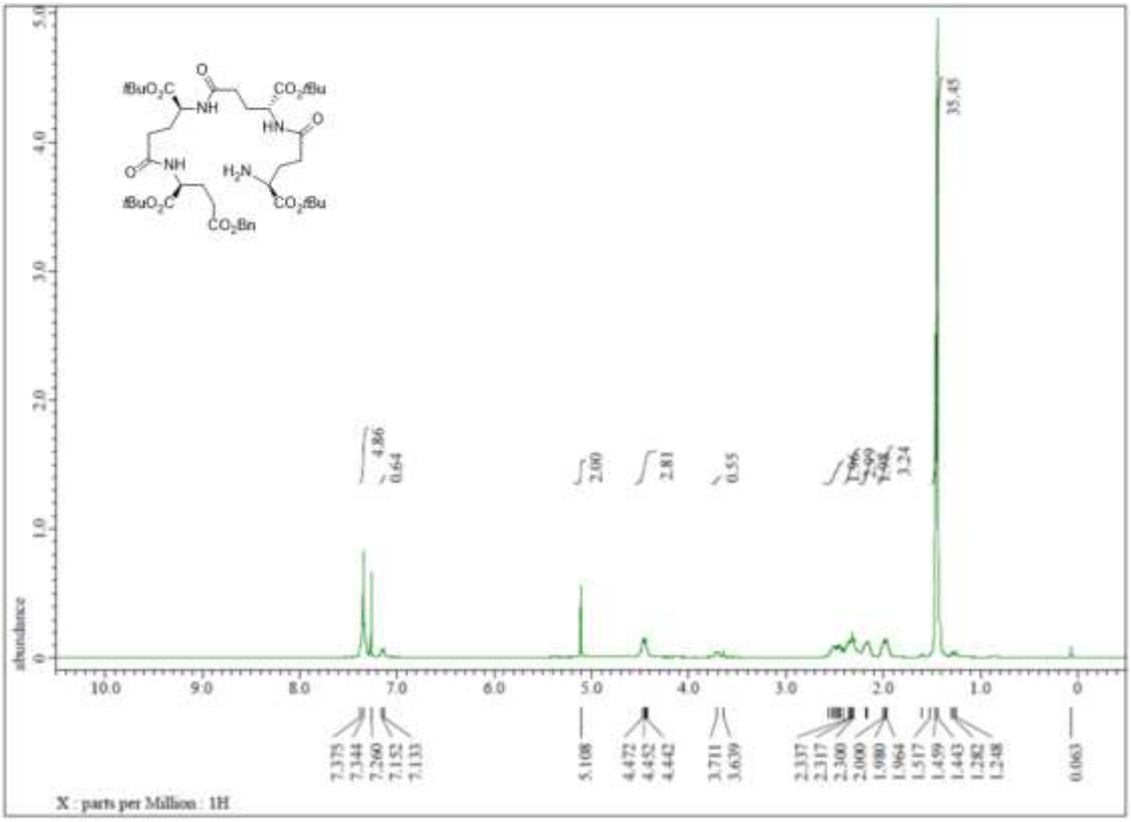

Figure S34. ^{1}H NMR ($CDCl_3$) spectrum for linear LLDL-glutamic acid with free amine.

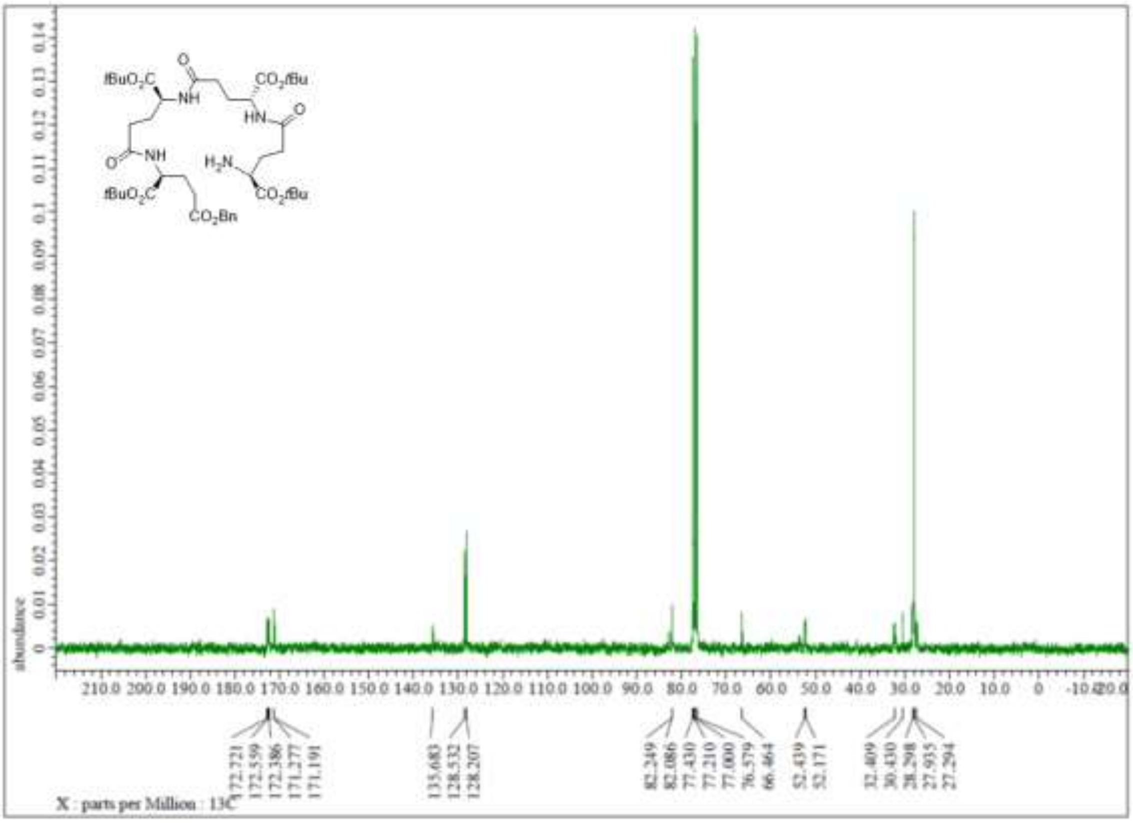

Figure S35. ^{13}C NMR ($CDCl_3$) spectrum for linear LLDL-glutamic acid with free amine.

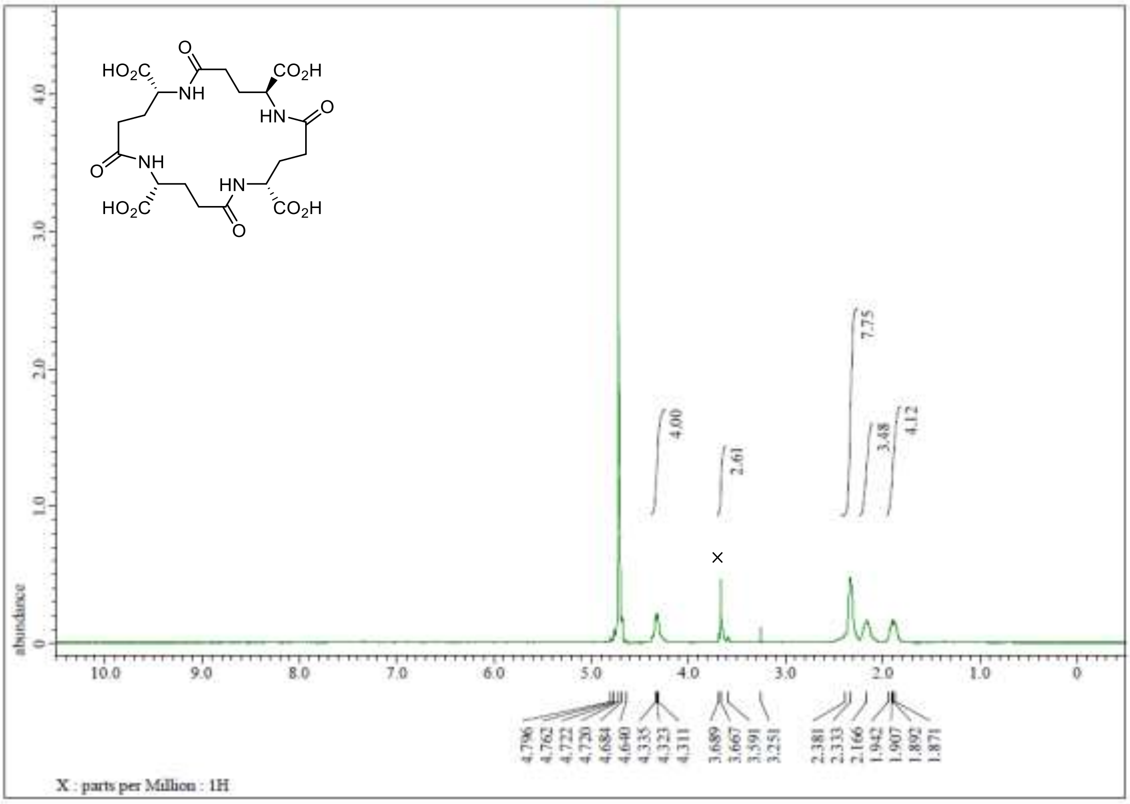

Figure S36. ^{1}H NMR (D_2O) spectrum for **2**.

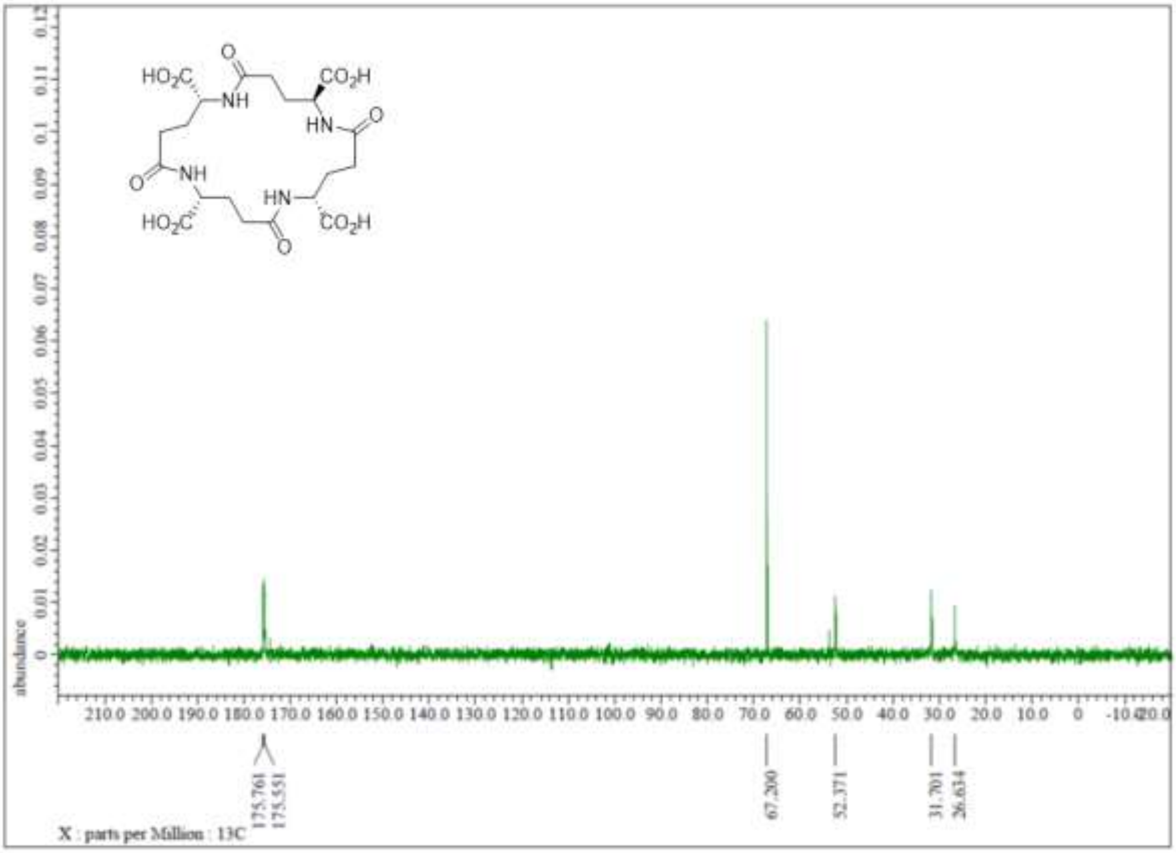

Figure S37. ^{13}C NMR (D_2O) spectrum for **2**; 1,4-dioxane added as internal standard.

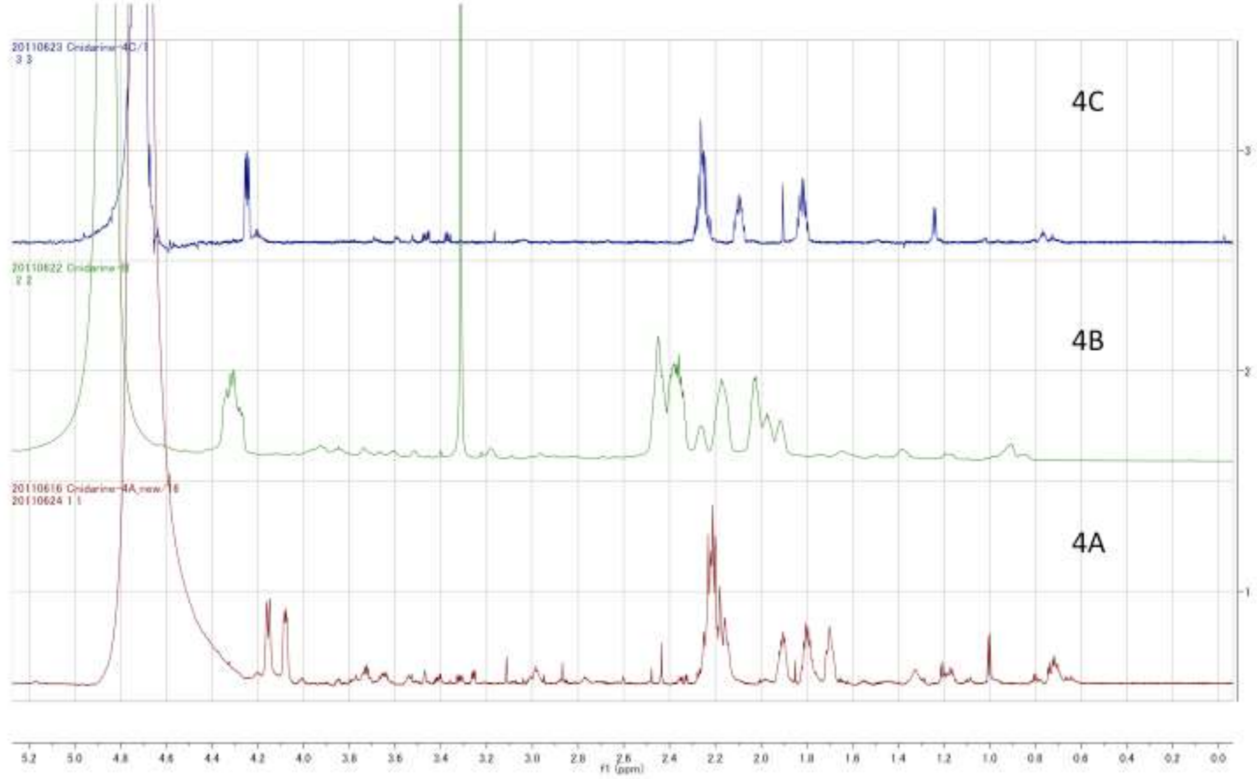

Figure S38. The ^{1}H NMR (800 MHz, D_2O with 10 μL HCOOH) spectra of isolated cnidarins 4A (**1**, bottom), 4B (**2**, middle), 4C (**3**, top).
